# Factors associated with cognitive impairment at 3, 6, and 12 months after the first stroke among Lebanese survivors

**DOI:** 10.1002/brb3.2837

**Published:** 2022-12-10

**Authors:** Celina F. Boutros, Walaa Khazaal, Maram Taliani, Najwane Said Sadier, Pascale Salameh, Hassan Hosseini

**Affiliations:** ^1^ Institut Mondor de Recherche Biomédicale (IMRB)‐Inserm U955, Ecole Doctorale Science de la Vie et de la Santé Université Paris‐Est Créteil Paris France; ^2^ Neuroscience Research Center Faculty of Medical Sciences Lebanese University Hadath Lebanon; ^3^ College of Health Sciences Abu Dhabi University Abu Dhabi United Arab Emirates; ^4^ Institut National de Santé Publique Epidémiologie Clinique et Toxicologie (INSPECT‐LB) Beirut Lebanon; ^5^ Faculty of Pharmacy Lebanese University Hadath Lebanon; ^6^ Department of Primary Care and Population Health University of Nicosia Medical School Nicosia Cyprus; ^7^ Neurology Department Hôpital Henri Mondor, AP‐HP Créteil France

**Keywords:** cognitive domains, complications, factors, follow‐up, Lebanon, MMSE, post‐stroke cognitive impairment, PSCI, rate, severity, survivors

## Abstract

**Introduction:**

This study aimed to calculate the rate of post‐stroke cognitive impairment (PSCI) by evaluating the cognitive domains among Lebanese stroke survivors at 3, 6, and 12 months post‐stroke, and to identify the contributing factors including pre‐ and post‐stroke related factors.

**Methods:**

A multicenter longitudinal prospective study was conducted in 10 hospitals from Beirut and Mount Lebanon for a 15‐month period. Mini‐Mental State Examination (MMSE), modified Rankin Scale (mRS), Short Form Health Survey (SF12), National Institutes of Health Stroke Scale (NIHSS), and Hospital Anxiety and Depression Scale (HADS) were used to assess cognitive function, disability degree, Quality of Life (QoL), stroke severity, and levels of anxiety and depression, respectively. Then, univariate and multivariable analyses were performed to identify the predictors of PSCI.

**Results:**

Low MMSE scores were found among survivors during the first 3 months post‐stroke (74.8%) of whom 53.7% presented with an MMSE ≤ 17, followed by 46.7% in the 6 months, and 37.6% at 12 months post‐stroke. Follow‐up comparisons showed a significant increase of MMSE scores over time (*p* < .001), indicating a 37% improvement of the cognitive function over time. The most affected cognitive domain was the attention and concentration at the three time points. Independent factors that were positively associated with low MMSE scores were as follows: sedentary behavior ≥ 12 h/day (AOR = 3.062, *p* = .033), involvement of the left hemisphere (AOR = 2.710, *p* = .006), HADS ≥ 11 (AOR = 2.536, *p* = .049), and high NIHSS scores (AOR = 3, *p* = .009). Age was the main predictor in the three time periods (AOR ≈ 3, *p* < .05). Inversely, female gender (AOR = 0.09, *p* = .027), high educational level (AOR = 0.2, *p* < .02), employment post‐stroke (AOR = 0.3, *p* = .023), high Physical Component Summary (PCS) of Quality of Life (QoL) (AOR = 0.8, *p* < .001), and the use of anti‐diabetic treatment post‐stroke (AOR = 0.17, *p* = .016) improved MMSE scores to > 23.

**Conclusion:**

The risk of PSCI among Lebanese stroke survivors was high especially in the acute phase, depending on various determinants. Health care providers are invited to implement an emergency rehabilitation program for an appropriate successful management of the risk factors in order to reduce stroke burden and to improve overall cognitive performance.

## Graphical abstract

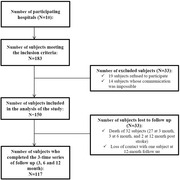

## 1 INTRODUCTION

Stroke is a widespread health concern affecting approximately 17 million people worldwide every year (Klamroth‐Marganska, 2018). The short‐term, medium‐term, and long‐term consequences of stroke are remarkable, with high mortality and morbidity rates requiring multidisciplinary care on a daily basis (Broussy et al., 2019).

Cognitive impairment following stroke is very common and can lead to dementia, placing an enormous burden on caregivers and the healthcare system (Rohde et al., 2019). The heterogeneous nature of cerebrovascular lesions may have an effect on cognition through various mechanisms including altered blood flow and oxygen supply, chronic inflammation, disruption of axonal tracts, or altered cortical connectivity. Stroke patients have a high potential to develop cognitive impairment within the first year of stroke onset starting from mild cognitive impairment (MCI) and ending with severe dementia (Al‐Qazzaz et al., 2014). According to the clinical diagnostic criteria and the Mini‐Mental State Examination (MMSE) test tool, MCI is defined by a cognitive decline, an MMSE score between 18 and 23, including four subtypes as follows: amnestic, amnestic plus other domains, nonamnestic single domain, and nonamnestic multiple domains, activities of daily living may be normal or mildly impaired; whereas dementia is a severe cognitive impairment, with an MMSE score ≤ 17, requiring a deficit in performance in ≥ 2 cognitive domains that are of sufficient severity to affect activities of daily living (Dichgans & Leys, 2017; Tombaugh & McIntyre, 1992). One or more cognitive domains may be altered, including attention and concentration, executive function, memory, language, and visuospatial cognitive domains (Ballard et al., 2003; Gorelick et al., 2011). However, the most impacted domains are the attention and executive function at various post‐stroke intervals (Cumming et al., 2013).

Two previous studies, in Norway and China, revealed that ≥60% of stroke survivors without pre‐existing cognitive disorder experienced post‐stroke cognitive impairment (PSCI) with MMSE ≤ 23 (Ihle‐Hansen et al., 2011; Qu et al., 2015). In Norway, 37.5% developed MCI (18≤ MMSE ≤ 23) versus 19.6% developed dementia (MMSE ≤ 17) within the first year post‐stroke (Ihle‐Hansen et al., 2011).

A systematic review conducted by Lo et al. in 2019 including 13 global studies showed that the highest proportion of PSCI in the 3 to 6 months post‐stroke was found in African Americans (48%) followed by whites (47%), Koreans (45%), Nigerians (40%), and Singaporean Chinese (35%) (Lo et al., 2019). Demographic characteristics, lifestyle, clinical parameters, and stroke‐related factors are classified as important contributors for PSCI (Hagberg et al., 2019; Kalaria et al., 2016; Lo et al., 2019; Mijajlović et al., 2017).

In Lebanon, stroke is identified as the second most common cause of death (El‐Hajj et al., 2016) due to the aging population, high rates of modifiable risk factors, and the low socio‐economic status (Gifford et al., 2022). Based on the countries’ classification of the World Bank (2021–2022), Lebanon is ranked among lower‐middle income countries in the Middle East and North Africa (MENA) region (World Bank, 2022). Although studies in Lebanon are still lacking regarding complications post‐stroke, stroke can be perceived as a high morbidity disease that highly burdens the country from all perspectives (Salhab et al., 2018). Several papers were previously published in Lebanon related to the stroke prevalence, risk factors, risk score, care practice, and mortality rate (El‐Hajj et al., 2019, 2016; Khazaal et al., 2021; Lahoud et al., 2018; Salameh et al., 2018; Salhab et al., 2018). However, so far, no studies in the MENA region, including Lebanon, have addressed the rate of PSCI and their predictors pre‐and post‐stroke conditions. This highlights the importance of our paper that aimed at studying the proportion of PSCI by evaluating the MMSE cognitive domains among stroke survivors in Lebanon and identifying the various contributing factors and characteristics—baseline socio‐demographics, pre‐existing conditions, lifestyle, and stroke‐related factors and complications at three time points, 3, 6, and 12 months after stroke.

## 2 MATERIALS AND METHODS

To ensure an adequate reporting of this work, we followed The Strengthening the Reporting of Observational studies in Epidemiology (STROBE) guidelines (von Elm et al., 2014).

### 2.1 Study design

This is an epidemiological, observational, multicenter, prospective, longitudinal study that was conducted in 10 hospitals from Beirut and Mount Lebanon (five university and five non‐university hospitals). It was conducted over a 15‐month period of follow‐up, starting from February 2018.

### 2.2 Participants

Participants were any Lebanese subject aged ≥ 18 years old admitted to the participating hospitals between February 2018 and May 2018, being a survivor of first‐time ischemic or hemorrhagic stroke identified by the following codes of the International Classification of Diseases‐10 (ICD‐10) (I63‐I61) (ICD‐10 Version:2016, 2016): cerebrovascular accident, stroke, ischemic stroke, hemorrhagic stroke, intracerebral hemorrhage, or embolic/cerebral vascular thrombosis. The diagnosis was confirmed with brain imaging and the clinical definition of the World Health Organization as follows: “it is a clinical syndrome consisting of rapidly developing clinical signs of focal (or global in case of coma) disturbance of cerebral function lasting more than 24 h or leading to death with no apparent cause other than a vascular origin” (Stroke Guidelines, 2016).

The exclusion criteria were the following: admission for a recurrent stroke or for transient ischemic accident or past medical history with neurologic disorders or cognitive sequelae (dementia, Alzheimer disease, ataxia, Bell's palsy, brain tumor, cerebral aneurysm, epilepsy, seizures, Parkinson disease, meningitis, hydrocephalus, encephalitis, aphasia, brain attack, etc.).

### 2.3 Sample size

Given that the estimated prevalence of stroke in Lebanon is 3.9% as reported by Jurjus et al. (2009), the minimum sample size was calculated using the Epi‐info 7 program to be 116 subjects. A sample size of 150 subjects was considered in the study taking into account those with incomplete data and others with loss to follow‐up.

### 2.4 Study procedures

Data collection was done by trained and qualified investigators to minimize errors. Hospitals were approached to secure approval after which eligible subjects are recruited. We selected the eligible subjects from different departments through an access to the paper or web‐based hospital patients’ registries. After that, a face‐to‐face interview with the subjects and their caregivers or legal representatives was essential to get their written consent and do the subsequent follow‐up home visits scheduled at 3, 6, and 12 months post‐stroke.

### 2.5 Questionnaire and scales

A structured standardized validated Arabic questionnaire was used. It was divided into five major sections: socio‐demographic characteristics (age, gender, place of residence, marital status, number of kids, age of subject's custodian, educational level of the subject and their custodian, employment status, number of household members, number of rooms, and type of health insurance), lifestyle (eating habits, practice of physical activity, alcohol and other substances consumption, and the social support), health indicators reported in the patients’ registries (anthropometric indices, family/medical/surgical history, comorbidities, treatment taken), disease and its severity (types/ stroke classification: Trial of ORG 10172 in Acute Stroke Treatment classification (TOAST) (Adams et al., 1993; Lindley et al., 1993)/location/ symptoms, length of hospital stay, severity of disease, degree of disability, evaluation of quality of life), and lastly, stroke consequences noticed at 3, 6, and 12 months (neuropsychiatric disorders, cognitive disorders, hyperglycemia, fatigue, post‐stroke pain, falls, pressure ulcers, pulmonary and urinary infections, deep vein thrombosis, pulmonary embolism, seizures, and recurrence of stroke).

### 2.6 Outcome measures

MMSE, first developed by Folstein in 1975 (Folstein et al., 1975), is widely used to study the post‐stroke cognition function. It measures seven cognitive domains, including orientation to time, orientation to place, memory registration, memory recall, attention/concentration, language, and visual construction. The cut‐off point is set at 24 defining a normal cognitive function. The severity has been classified into three levels: 24–30 = no cognitive impairment; 18–23 = mild cognitive impairment; and 0–17 = severe cognitive impairment (Tombaugh & McIntyre, 1992) (Cronbach's alpha of (*r*) = .882). Recent research has validated the use of the Arabic version of MMSE among the Lebanese population. Intra‐rater and inter‐rater test–retest score correlations were 0.89 and 0.72, respectively. A sensitivity of 80% and a specificity of 89.4% were provided (El‐Hayeck et al., 2019).

National Institute of Health Stroke Scale (NIHSS) is a measurement tool that was created in 1989 by Brott T. and his colleagues (Brott et al., 1989), and reported as the most reliable and valid stroke severity measuring instrument (Young et al., 2005). It is a scale that includes 15 elements for the evaluation of consciousness, language, motor function, sensory loss, visual fields, extra‐ocular movements, coordination, neglect, and speech. It is divided into five levels: 0 = no stroke, 1–4 = minor stroke, 5–14 = moderate stroke, 15–20 = moderate to severe stroke, and 21–42 = severe stroke (FAOTA, 2015) (Cronbach's alpha of (*r*) = .942). We used the validated Arabic version of NIHSS, with intra‐rater and inter‐rater agreements of 0.94 and 0.95, respectively (Hussein et al., 2015).

Modified Rankin Scale (mRS), initially described by Rankin J. in 1957 (Rankin, 1957), is a scale to evaluate the degree of disability in stroke survivors. This scale is one of the most used tools in clinical trials, given its reliability (Kappa = 0.81), validity, and ability to distinguish between relevant disability levels and recovery status (Banks & Marotta, 2007; Cheng et al., 2014). mRS is divided into seven levels from 0 (no symptoms) to 6 (death). The mild disability (independence) is graded 0 to 2 and the moderate to severe disability is graded ≥ 3 (Modified Rankin Scale, 2017) (Cronbach's alpha of (*r*) = .946).

Quality of life, Short form Health survey (SF12), is a questionnaire developed by Ware et al. in 1996 (Ware et al., 1996) and divided into two summary scores: physical and mental component summaries (PCS and MCS). They express the mental and physical functions and overall health‐related‐quality of life. PCS and MCS are computed through the scores of 12 questions and range from 0 (lowest level of health) to 100 (highest level of health) (Cronbach's alpha of (*r*) = .540). The validated Arabic translation was considered (Al‐Shehri et al., 2008; Sabbah et al., 2003).

Questionnaire for Verifying Stroke‐Free Status, designed by Meschia et al. in 2000 (Meschia et al., 2000), is an eight‐item questionnaire tackling the stroke symptoms. The purpose of this questionnaire is to verify stroke‐free status of the subjects; however, in our study which enrolled only confirmed stroke cases, the purpose was to report the symptoms experienced by survivors following a defined, structured, and valid questionnaire.

Social Support Rating Scale, developed by Xiao in 1994 (Xiao, 1994), is an instrument of 10 items to measure three dimensions of the social support. Item scores are simply summed up, generating a total support score ranging from 12 to 66. This total support score is classified into three categories: low (≤ 22), moderate (23–44), and high (≥ 45).

Hospital Anxiety and Depression Scale (HADS), designed by Zigmond and Snaith in 1983 (Zigmond & Snaith, 1983), measures the levels of anxiety and depression. It is a 14‐item questionnaire, widely used, divided into two scales: a scale for depression and a scale for anxiety. Scores range from 0 to 7 = normal, 8 to 10 = borderline, and 11 to 21 = abnormal (Vansimaeys et al., 2017). A recent validated Arabic version of HADS scale was published by Al Aseri et al. in 2020 (Al Aseri et al., 2020) (Cronbach's alpha of (*r*) = .906).

Fatigue severity scale (FSS) is a nine‐item self‐report questionnaire scale developed by Krupp et al. in 1989 (Krupp et al., 1989). It is the most commonly used tool to investigate the severity of fatigue in different contexts. Each item is classified from 1 (strong disagreement) to 7 (strong agreement). The cut‐off was set to be ≥ 4 (Rosti‐Otajärvi et al., 2017; Valko et al., 2008). The validated Arabic version was used (Al‐Sobayel et al., 2016) (Cronbach's alpha of (*r*) = .854).

Visual analogue scale (VAS), first introduced in 1921 (Hayes & Patterson, 1921), is used to express the value of the total impression for the severity of pain by making a mark determining an integer number from 0 to 10. The cut‐off was set to be ≥ 4 (Cronbach's alpha of (*r*) = .866).

Douleur Neuropathique 4 (DN4) is a 4‐item questionnaire developed by Bouhassira and colleagues in 2005 (Bouhassira et al., 2005). It is a reliable scale that differentiates between neuropathic pain and non‐neuropathic pain. For scoring, 1 is given to each positive and 0 to each negative item (total score range 0–10). The cut‐off value for diagnosis of neuropathic pain is a total score of ≥ 4 (Cronbach's alpha of (*r*) = .878). A validated Arabic version was used (Terkawi et al., 2017).

Modified Ashworth scale (MAS), described by Bohannon and Smith in 1987 (Bohannon & Smith, 1987), is an easy scale and commonly used in clinical practice for the measurement and classification of spasticity. This scale grades the muscle tone from 0 (normal) to 4 (severe spasticity). The cut‐off value for diagnosis of spasticity is a total score of ≥ 3 (Cao et al., 2022) (Cronbach's alpha of (*r*) = .933).

### 2.7 Data processing and analysis

Collected data were coded, introduced, and entered into the software Statistical Package for Social Sciences (SPSS) version 25 (**SPSS^TM^ Inc**., Chicago, IL, USA). First, descriptive analyses were performed using numbers and percentages for qualitative variables and means with standard deviation (SD) for continuous variables. In addition, a one way repeated measure Analysis of Variances (ANOVA) (Bonferroni method) was important to assess the MMSE scores over time. Second, the univariate analyses were conducted. The student's *t*‐test and one way ANOVA were used for the comparison of means of quantitative variables with a normal distribution and equal variances, whereas the non‐parametric (Kruskal–Wallis test) test for quantitative variables with an abnormal distribution and the unequal variances were used. The Chi‐square test was used for the comparison of percentages between two qualitative variables. Then, multivariable analysis using multinomial logistic regressions adjusted for age, gender, and education was made to examine independent determinants of mild and severe cognitive impairment. The variables with univariate association at *p* ≤ .05 were entered into logistic regression. The goodness‐of‐fit statistic is reported to determine if the model provides a good fit for the data (*p* > .05). The strength of association was interpreted using the adjusted odds ratio (AOR) with 95% confidence interval (CI). A *p*‐value ≤ .05 was considered statistically significant.

## 3 RESULTS

**FIGURE 1:**
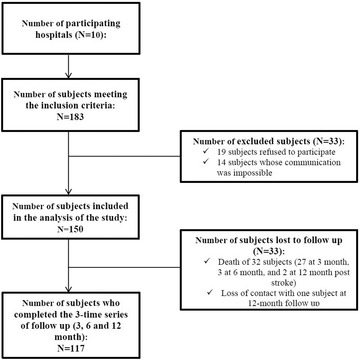
Flow diagram of the steps followed to obtain the sample of the study

A total of 10 hospitals agreed to participate in the study. Out of 183 subjects who met the required inclusion criteria, 150 were recruited in the study (82% of the response rate), of whom 117 completed the whole period of follow‐up (shown in Figure 1).

### 3.1 Baseline characteristics of the study participants

In total, 88 (58.7%) were males and 62 (41.3%) were females. The mean age was 73.7 with SD of 12 years. The majority were married (78%), lived with family members (96.1%) and 87% in less crowded houses. An important proportion of the participants were illiterate (38%). In addition, 67.3% were without any profession or retired. On the other hand, 20% of the subjects did not resume their work after having the stroke incident (Table 1).

**TABLE 1:** Baseline characteristics of the study population

Variables	Frequency (*N*)	Percentage (%)	Mean (± SD)
**Gender** (*N* = 150)			
Male	88	58.7	
Female	62	41.3	
**Age** (*N* = 150)			
30–39 years	1	0.7	
40–49 years	7	4.7	
50–59 years	15	10.0	
60–69 years	29	19.3	73.73 (± 12.08)
70 – 79 years	45	30.0	
80–89 years	46	30.7	
90–99 years	7	4.7	
**Marital status** (*N* = 150)			
Single	14	9.3	
Married	117	78.0	
Divorced	2	1.3	
Widowed	17	11.3	
**Family members** (*N* = 128)			
Alone	5	3.9	
With family members	123	96.1	
**Residence** (*N* = 150)			
Beirut	33	22.0	
Mount Lebanon	107	71.3	
Bekaa	3	2.0	
North Lebanon	2	1.3	
South Lebanon	5	3.3	
**Education level** (*N* = 150)			
Illiterate	57	38.0	
Primary/Complementary	47	31.3	
Secondary	19	12.7	
University	27	18.0	
**Employment status post‐stroke** (*N* = 150)			
Person without any profession/ retired	101	67.3	
Unemployed	30	20.0	
Employed	19	12.7	
**Social Security** (*N* = 150)			
No	25	16.7	
Yes	125	83.3	
**Presence of a caregiver** (*N* = 150)			
No	98	65.3	
Yes	52	34.7	
**Caregiver's age** (*N* = 52)			
Adult: 20 – 40 years	9	17.3	
Middle age: 40 – 60 years	35	67.3	
Elderly: > 60 years	8	15.4	
**Stroke types**			
Hemorrhagic stroke (ICH)	7	4.7	
Ischemic stroke	143	95.3	
TOAST Classification			
LAA	58	45.7	
CE	6	4.7	
SVO	63	49.6	
OE	0	0.0	
UE	0	0.0	
**Stroke location**			
Right hemisphere	60	40.0	
Left hemisphere	70	46.7	
Bilateral hemisphere	10	6.7	
Brainstem	1	0.7	
Cerebellum	9	6.0	

Abbreviations: SD, standard deviation; ICH, intracerebral hemorrhage; TOAST, Trial of ORG 10172 in Acute Stroke Treatment; LAA, large artery atherosclerosis; CE, cardioembolism; SVO, small vessel occlusion; OE, stroke of other determined etiology; UE, stroke of undetermined etiology.

### 3.2 Stroke characteristics and its severity

As shown in Table 1, a total of 143 subjects (95.3%) suffered from ischemic stroke classified as follows: 48.8% with small vessel occlusion (SVO), 46.3% with large artery atherosclerosis (LAA), and 5% with cardioembolism (CE), versus 7 subjects (4.7%) who suffered from intracerebral hemorrhagic stroke. Regarding the affected location, 46.7% were found to have involvement of the left hemisphere while 40% of the subjects had right hemisphere involvement. There were only 6.8% of subjects who had received an intravenous thrombolysis.

The majority (70%) was not able to express themselves verbally or in writing at the time of stroke and experienced a weakness on one side of the body. The stroke severity, degree of disability, and components of QoL were estimated by NIHSS scores, mRS scores, and SF12 scores, respectively, at each time of the follow‐up. At 3 months, 16.8% of the subjects were found to have severe stroke (Figure S1). mRS scores were decreasing over time indicating greater independence in ADL, ranging from 16% to 18% with scores ≥ 5 at 3 months to less than 1.6% at 12 months post‐stroke (Figure S2). Regarding SF12 scores summarized in Table S1, we obtained low scores of physical (PCS) and mental (MCS) components of QoL (means between 28 and 40) at 3, 6, and 12 months. Levels were less than the theoretical average scores (cut‐off of 50 for PCS and 42 or MCS). At index admission, 47 (31.3%) subjects were already on antiplatelet and anticoagulation agents. At discharge, these drugs were prescribed to 141 (94%) subjects.

### 3.3 Rate of PSCI

Out of 150 enrolled subjects, 27 were deceased (18%) in the 3 months post‐stroke, followed by 3 (2.4%) in the 6 months and 2 (1.7%) in the 12 months of follow‐up. One subject was lost to follow up by the 12‐month visit. Therefore, we could not have their MMSE.

**FIGURE 2:**
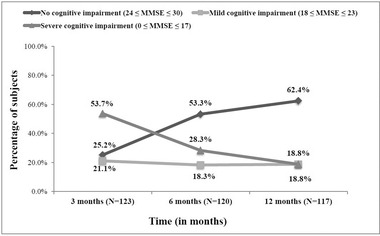
Severity of cognitive impairment over 3, 6, and 12 months post‐stroke. MMSE, Mini‐Mental State Examination

**FIGURE 3:**
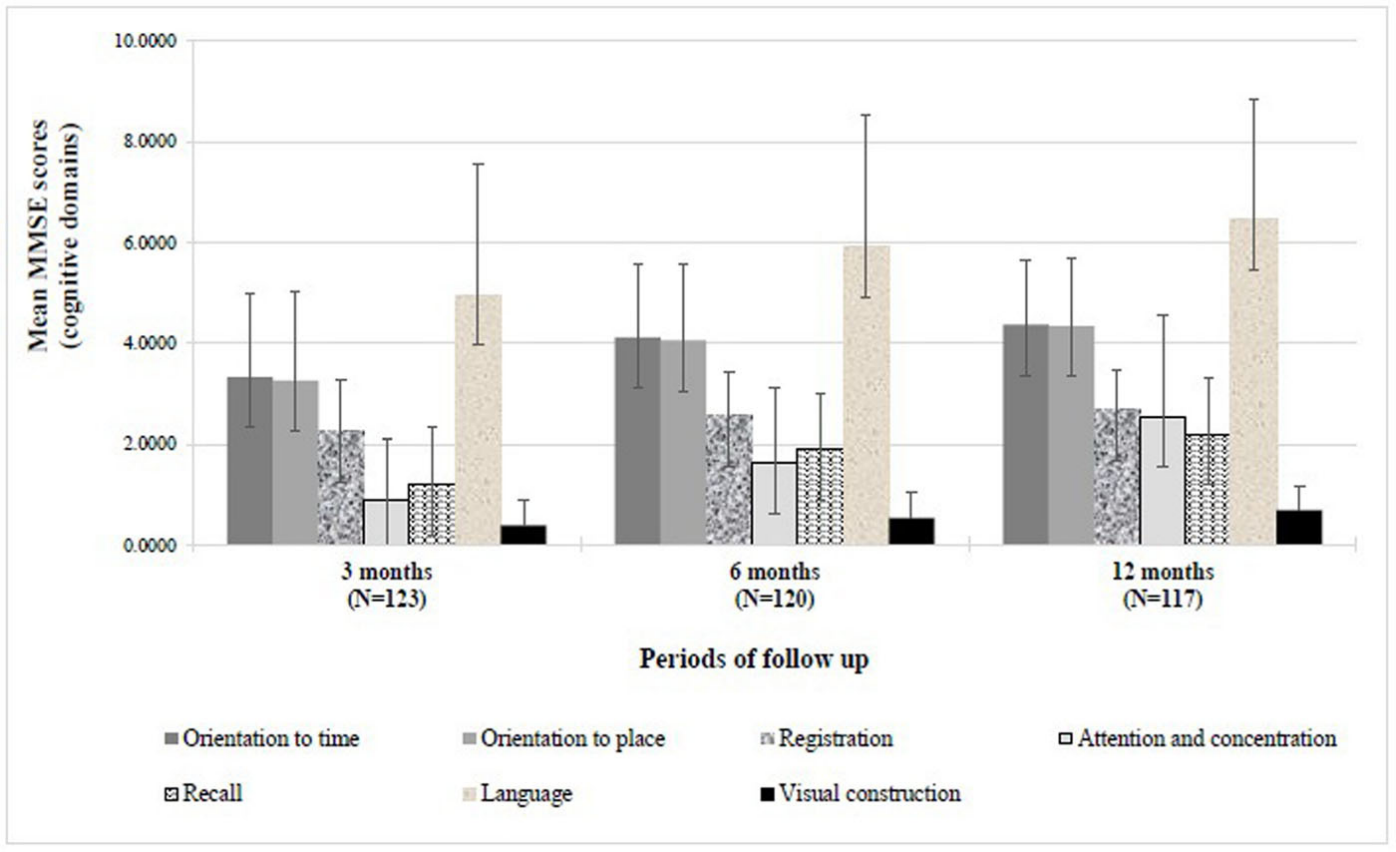
Mean scores of the different cognitive domains of MMSE across the three periods of follow‐up

At 3 months post‐stroke, the majority of stroke survivors (74.8%, *n* = 123) were cognitively impaired of whom 66 (53.7%) with severe impairment (MMSE ≤ 17) and 26 (21.1%) with mild impairment (18 ≤ MMSE < 24), whereas 31 survivors (25.2%) remained cognitively intact (MMSE ≥ 24). The cognitive impairment gradually decreased over time, at 6 and 12 months post‐stroke, with percentages of 46.7% (28.3% severely impaired and 18.3% mildly impaired) and 37.6% (18.8% with mild to severe cognitive impairment), respectively (shown in Figure 2 and Figure S3). Figure 3 and Figure S4 summarize the different cognitive domain scores of MMSE across the three time series. We found that all cognitive domains were affected in the 3‐month post‐stroke with the highest percentage of impairment detected in attention/concentration (96.7%, score < 4) and memory recall (82.9%, score < 3), followed by the visual construction (61%, score = 0), orientation to time and place (50%, score < 4), language (43.1%, score < 5), and memory registration (41.5%, score < 3). A substantial improvement of these domains was highlighted in the following 6 to 12 months post‐stroke, except for the impairment of attention/concentration, which slightly decreased from 10% to 20% but remained to be of particular concern (85% at 6 months vs 63.2% at 12 months).

A one way repeated measures ANOVA was conducted to evaluate the null hypothesis that there was no change in the cognitive function over the time after the first‐stroke (*N* = 117). The results designated a significant time effect, Wilks’ Lambda = 0.528, *F* (2, 115) = 51.439, *p* < .001, *n* ^2^ = 0.472. Thus, there was evidence to reject the null hypothesis. Follow‐up comparisons indicated that each pairwise difference was significant, *p* < .001. There was a significant increase of MMSE scores over time, indicating an improvement of the cognitive function over time.

### 3.4 Factors associated with cognitive impairment post‐stroke

Univariate analysis was made to compare the subjects with PSCI and the unharmed subjects, at 3, 6, and 12 months (Tables 2, 3, 4 and Tables S2–S, 4).

**TABLE 2:** The association between socio‐demographic characteristics and the post‐stroke cognitive impairment among Lebanese stroke survivors

Socio‐demographic characteristics	3 months post‐stroke				6 months post‐stroke				12 months post‐stroke			
	No PSCI *N* (%)	Mild PSCI *N* (%)	Severe PSCI*N* (%)	*p*‐Value	No PSCI *N* (%)	Mild PSCI *N* (%)	Severe PSCI *N* (%)	*p*‐Value	No PSCI*N* (%)	Mild PSCI *N* (%)	Severe PSCI *N* (%)	*p*‐Value
**Gender**												
Male	22 (71.0)	16 (61.5)	37 (56.1)	.373	39 (60.9)	13 (59.1)	21 (61.8)	0.980	43 (58.9)	13 (59.1)	14 (63.6)	.921
Female	9 (29.0)	10 (38.5)	29 (43.9)		25 (39.1)	9 (40.9)	13 (38.2)		30 (41.1)	9 (40.9)	8 (36.4)	
**Age**												
< 65 years old	15 (48.4)	7 (26.9)	12 (18.2)	**.008**	23 (35.9)	5 (22.7)	6 (17.6)	0.130	26 (35.6)	4 (18.2)	2 (9.1)	**.028**
≥ 65 years old	16 (51.6)	19 (73.1)	54 (81.8)		41 (64.1)	17 (77.3)	28 (82.4)		47 (64.4)	18 (81.8)	20 (90.9)	
**Educational level**												
Illiterate or Primary/Complementary	16 (51.6)	17 (65.4)	49 (74.2)	.087	33 (51.6)	20 (90.9)	26 (76.5)	**0.001**	40 (54.8)	19 (86.4)	18 (81.8)	**.005**
Secondary or University	15 (48.4)	9 (34.6)	17 (25.8)		31 (48.4)	2 (9.1)	8 (23.5)		33 (45.2)	3 (13.6)	4 (18.2)	
**Employment status post‐stroke**												
Person without any profession or retired	10 (32.3)	20 (76.9)	48 (72.7)	< .001*	38 (59.4)	15 (68.2)	23 (67.6)	0.073*	45 (61.6)	17 (77.3)	13 (59.1)	.063*
Unemployed	7 (22.6)	3 (11.5)	16 (24.2)		11 (17.2)	4 (18.2)	10 (29.4)		12 (16.4)	4 (18.2)	8 (36.4)	
Employed	14 (45.2)	3 (11.5)	2 (3.0)		15 (23.4)	3 (13.6)	1 (2.9)		16 (21.9)	1 (4.5)	1 (4.5)	
**Presence of a caregiver**												
No	27 (87.1)	17 (65.4)	35 (53.0)	**.005**	46 (71.9)	14 (63.6)	19 (55.9)	0.275	52 (71.2)	13 (59.1)	13 (59.1)	.402
Yes	4 (12.9)	9 (34.6)	31 (47.0)		18 (28.1)	8 (36.4)	15 (44.1)		21 (28.8)	9 (40.9)	9 (40.9)	
**HCI**												
*≤ 1*	29 (93.5)	22 (84.6)	57 (86.4)	.572*	60 (93.8)	18 (81.8)	28 (82.4)	0.115*	68 (93.2)	17 (77.3)	18 (81.8)	.071*
*>1*	2 (6.5)	4 (15.4)	9 (13.6)		4 (6.3)	4 (18.2)	6 (17.6)		5 (6.8)	5 (22.7)	4 (18.2)	

Abbreviations: HCI, Household Crowding Index PSCI, Post‐Stroke Cognitive Impairment: It assesses the socio‐economic level, defined by the number of household members (excluding new‐borns) divided by the number of rooms. It has 2 levels: ≤ 1, that is, maximum one person per room reflecting a house less crowded; > 1, that is, more than one person per room reflecting a house too crowded.

**TABLE 3:** The association between stroke characteristics and the post‐stroke cognitive impairment among Lebanese stroke survivors

Stroke characteristics and its severity	3 months post‐stroke				6 months post‐stroke				12 months post‐stroke			
	No PSCI *N* (%) or Mean (±SD)	Mild PSCI *N* (%) or Mean (±SD)	Severe PSCI *N* (%) or Mean (±SD)	*p*‐value	No PSCI *N* (%) or Mean (±SD)	Mild PSCI *N* (%) or Mean (±SD)	Severe PSCI *N* (%) or Mean (±SD)	*p*‐value	No PSCI *N* (%) or Mean (±SD)	Mild PSCI *N* (%) or Mean (±SD)	Severe PSCI *N* (%) or Mean (±SD)	*p*‐value
**Duration between symptoms onset and admission to hospital**												
≤ 3 h	19 (61.3)	24 (92.3)	55 (83.3)	.433	51 (79.7)	18 (81.8)	26 (76.5)	.931*	56 (76.7)	19 (86.4)	17 (77.3)	.794*
3 to 6 h	5 (16.1)	0 (0.0)	5 (7.6)		5 (7.8)	1 (4.5)	4 (11.8)		6 (8.2)	1 (4.5)	3 (13.6)	
≥ 6 h	7 (22.6)	2 (7.7)	6 (9.1)		8 (12.5)	3 (13.6)	4 (11.8)		11 (15.1)	2 (9.1)	2 (9.1)	
**Intravenous thrombolysis**												
No	25 (96.2)	22 (91.7)	53 (93.0)	.882*	53 (93.0)	19 (95.0)	26 (92.9)	1.000*	61 (91.0)	17 (94.4)	19 (100)	.539*
Yes	1 (3.8)	2 (8.3)	4 (7.0)		4 (7.0)	1 (5.0)	2 (7.1)		6 (9.0)	1 (5.6)	0 (0.0)	
**Duration of hospital stay (days)**	5.77 (± 4.51)	10 (± 8.26)	9.77 (± 8.90)	**.001^‡^**	6.59 (± 4.41)	10.82 (± 9.54)	11.82 (± 10.94)	**.004**	7.92 (± 7.73)	9.32 (± 5.59)	10.32 (± 9.05)	.060^‡^
**Stroke types**												
Haemorrhagic stroke	0 (0.0)	3 (11.5)	2 (3.0)		3 (4.7)	0 (0.0)	2 (5.9)		3 (4.1)	0 (0.0)	2 (9.1)	
Ischemic stroke	31 (100)	23 (88.5)	64 (97.0)	.084*	61 (95.3)	22 (100)	32 (94.1)	.706*	70 (95.9)	22 (100)	20 (90.9)	.445*
TOAST Classification												
LAA	11 (42.3)	7 (33.3)	22 (40.0)	.731*	20 (37.0)	5 (25.0)	14 (53.8)	.287*	26 (40.6)	4 (22.2)	8 (47.1)	.386*
CE	0 (0.0)	1 (4.8)	4 (7.3)		2 (3.7)	1 (5.0)	1 (3.8)		2 (3.1)	1 (5.6)	1 (5.9)	
SVO	15 (57.7)	13 (61.9)	29 (52.7)		32 (59.3)	14 (70.0)	11 (42.3)		36 (56.3)	13 (72.2)	8 (47.1)	
**Stroke location**												
Right hemisphere	13 (41.9)	13 (50.0)	21 (31.8)	.129*	25 (39.1)	10 (45.5)	12 (35.3)	.186*	28 (38.4)	10 (45.5)	7 (31.8)	.006*
Left hemisphere	16 (51.6)	9 (34.6)	33 (50.0)		34 (53.1)	7 (31.8)	15 (44.1)		40 (54.8)	5 (22.7)	10 (45.5)	
Bilateral hemisphere	1 (3.2)	0 (0.0)	8 (12.1)		1 (1.6)	3 (13.6)	4 (11.8)		1 (1.4)	3 (13.6)	4 (18.2)	
Brainstem	0 (0.0)	1 (3.8)	0 (0.0)		1 (1.6)	0 (0.0)	0 (0.0)		0 (0.0)	1 (4.5)	0 (0.0)	
Cerebellum	1 (3.2)	3 (11.5)	4 (6.1)		3 (4.7)	2 (9.1)	3 (8.8)		4 (5.5)	3 (13.6)	1 (4.5)	
**Stroke severity at 3 months post‐stroke**												
NIHSS < 21	31 (100)	26 (100)	45 (68.2)	< .001*	64 (100)	20 (90.9)	16 (47.1)	< .001*	70 (95.9)	19 (86.4)	9 (40.9)	< .001*
NIHSS ≥ 21	0 (0.0)	0 (0.0)	21 (31.8)		0 (0.0)	2 (9.1)	18 (52.9)		3 (4.1)	3 (13.6)	13 (59.1)	
**Stroke severity at 6 months post‐stroke**												
NIHSS < 21					64 (100)	22 (100)	23 (67.6)	< .001*	71 (97.3)	22 (100)	14 (63.6)	< .001*
NIHSS ≥ 21					0 (0.0)	0 (0.0)	11 (32.4)		2 (2.7)	0 (0.0)	8 (36.4)	
**Stroke severity at 12 months post‐stroke**												
NIHSS < 21									72 (100)	22 (100)	15 (68.2)	< .001*
NIHSS ≥ 21									0 (0.0)	0 (0.0)	7 (31.8)	
**Degree of disability at 3 months post‐stroke**												
mRS < 3	25 (80.6)	11 (42.3)	10 (15.2)	**< .001**	41 (64.1)	3 (13.6)	2 (5.9)	**< .001**	39 (53.4)	5 (22.7)	1 (4.5)	**< .001**
mRS ≥ 3	6 (19.4)	15 (57.7)	56 (84.8)		23 (35.9)	19 (86.4)	32 (94.1)		34 (46.6)	17 (77.3)	21 (95.5)	
**Degree of disability at 6 months post‐stroke**												
mRS < 3					53 (82.8)	8 (36.4)	4 (11.8)	**< .001**	52 (71.2)	10 (45.5)	2 (9.1)	**< .001**
mRS ≥ 3					11 (17.2)	14 (63.6)	30 (88.2)		21 (28.8)	12 (54.5)	20 (90.9)	
**Degree of disability at 12 months post‐stroke**												
mRS < 3									60 (82.2)	13 (59.1)	3 (13.6)	**< .001**
mRS ≥ 3									13 (17.8)	9 (40.9)	19 (86.4)	
**Quality of life (SF‐12) 3 months post‐stroke**												
PCS	36.28 (± 7.88)	29.24 (± 6.19)	25.57 (± 4.52)	**< .001**	31.29 (± 8.2)	28.02 (± 5.63)	25.99 (± 5.2)	**.002**	30.75 (± 8.14)	26.84 (± 4.14)	26.57 (± 5.63)	.052
MCS	37.96 (± 9.24)	32.76 (± 9.67)	30.56 (± 8.32)	**.001**	35.18 (± 9.81)	33.93 (± 8.46)	28.87 (± 7.11)	**.003**	34.53 (± 9.33)	33.95 (± 9.07)	28.04 (± 7.44)	**.011**
**Quality of life (SF‐12) 6 months post‐stroke**												
PCS					38.56 (± 9.48)	32.47 (± 6.37)	29.65 (± 7.06)	**< .001**	37.13 (± 9.39)	33.24 (±7.52)	29.49 (± 6.58)	**.001**
MCS					37.99 (± 10.55)	36.4 (± 9.61)	29.05 (± 8.18)	**< .001**	37.43 (± 10.33)	35.5 (± 10.94)	27.99 (± 6.93)	**< .001**
**Quality of life (SF‐12) 12 months post‐stroke**												
PCS									43.86 (± 10.59)	34.04 (± 7.85)	29.98 (± 8.15)	**< .001**
MCS									44.22 (± 12.27)	37.08 (± 10.57)	29.11 (± 9.43)	**< .001**

Abbreviations: SD, Standard deviation; LAA, large artery atherosclerosis; CE, cardioembolism; SVO, small vessel occlusion; NIHSS, National Institutes of Health Severity Scale; mRS, Modified Rankin Scale; SF‐12, Short Form Health Survey‐12; PCS, Physical Component Summary; MCS, Mental Component Summary TOAST, Trial of Org 10172 in Acute Stroke Treatment; PSCI, Post‐Stroke Cognitive Impairment.

^‡^Kruskal–Wallis test was used when the statistical assumption of homogeneity of variance has been not met.

**TABLE 4:** The association between complications post‐stroke and post‐stroke cognitive impairment among Lebanese stroke survivors

**Complications post‐stroke**	**3 months post‐stroke**				**6 months post‐stroke**				**6 months post‐stroke**			
	**No PSCI *N* (%)**	**Mild PSCI *N* (%)**	**Severe PSCI *N* (%)**	***p*‐Value**	**No PSCI *N* (%)**	**Mild PSCI *N* (%)**	**Severe PSCI *N* (%)**	***p*‐Value**	**No PSCI *N* (%)**	**Mild PSCI *N* (%)**	**Severe PSCI *N* (%)**	***p*‐Value**
**Anxiety 3 months post‐stroke**												
HADS_A ≤ 7	19 (61.3)	10 (38.5)	31 (47.0)	**.022**	43 (67.2)	7 (31.8)	10 (29.4)	**.002**	45 (61.6)	7 (31.8)	6 (27.3)	.014*
8 ≤ HADS_A ≤ 10	8 (25.8)	2 (7.7)	12 (18.2)		8 (12.5)	6 (27.3)	8 (23.5)		11 (15.1)	6 (22.7)	5 (22.7)	
HADS_A ≥ 11	4 (12.9)	14 (53.8)	23 (34.8)		13 (20.3)	9 (40.9)	16 (47.1)		17 (23.3)	9 (40.9)	11 (50.0)	
**Anxiety 6 months post‐stroke**												
HADS_A ≤ 7					42 (65.6)	10 (45.5)	10 (29.4)	**.007**	45 (61.6)	9 (40.9)	6 (27.3)	.008*
8≤HADS_A ≤ 10					8 (12.5)	6 (27.3)	7 (20.6)		10 (13.7)	7 (31.8)	3 (13.6)	
HADS_A ≥ 11					14 (21.9)	6 (27.3)	17 (50.0)		18 (24.7)	6 (27.3)	13 (59.1)	
**Anxiety 12 months post‐stroke**												
HADS_A ≤ 7									57 (78.1)	10 (45.5)	6 (30.0)	< .001*
8≤HADS_A ≤ 10									6 (8.2)	7 (31.8)	3 (15.0)	
HADS_A ≥ 11									10 (13.7)	5 (22.7)	11 (55.0)	
**Depression 3 months post‐stroke**												
HADS_D ≤ 7	14 (45.2)	5 (19.2)	9 (13.6)	**< .001**	25 (39.1)	1 (4.5)	2 (5.9)	**< .001**	25 (34.2)	1 (4.5)	1 (4.5)	< .001*
8≤HADS_D ≤ 10	10 (32.3)	5 (19.2)	5 (7.6)		14 (21.9)	3 (13.6)	2 (5.9)		15 (20.5)	4 (18.2)	0 (0.0)	
HADS_D ≥ 11	7 (22.6)	16 (61.5)	52 (78.8)		25 (39.1)	18 (81.8)	30 (88.2)		33 (45.2)	17 (77.3)	21 (95.5)	
**Depression 6 months post‐stroke**												
HADS_D ≤ 7					27 (42.2)	2 (9.1)	2 (5.9)	**< .001**	27 (37.0)	2 (9.1)	1 (4.5)	< .001*
8≤HADS_D ≤ 10					14 (21.9)	4 (18.2)	3 (8.8)		15 (20.5)	5 (22.7)	1 (4.5)	
HADS_D ≥ 11					23 (35.9)	16 (72.7)	29 (85.3)		31 (42.5)	15 (68.2)	20 (90.9)	
**Depression 12 months post‐stroke**												
HADS_D ≤ 7									44 (60.3)	4 (18.2)	2 (10.0)	< .001*
8 ≤ HADS_D ≤ 10									12 (16.4)	3 (13.6)	1 (5.0)	
HADS_D ≥ 11									17 (23.3)	15 (68.2)	17 (85.0)	
**Falls 3 months post‐stroke**												
No falls	28 (90.3)	17 (65.4)	32 (48.5)	**< .001**	49 (76.6)	12 (54.5)	14 (41.2)	**.002**	54 (74.0)	11 (50.0)	8 (36.4)	**.003**
At least 1 fall	3 (9.7)	9 (34.6)	34 (51.5)		15 (23.4)	10 (45.5)	20 (58.8)		19 (26.0)	11 (50.0)	14 (63.6)	
**Falls 6 months post‐stroke**												
No falls					59 (92.2)	17 (77.3)	22 (64.7)	**.003**	65 (89.0)	17 (77.3)	13 (59.1)	.006*
At least 1 fall					5 (7.8)	5 (22.7)	12 (35.3)		8 (11.0)	5 (22.7)	9 (40.9)	
**Epileptic seizures 6 months post‐stroke**												
No					64 (100)	20 (90.9)	28 (82.4)	.001*	70 (95.9)	20 (90.9)	19 (86.4)	.174*
Yes					0 (0.0)	2 (9.1)	6 (17.6)		3 (4.1)	2 (9.1)	3 (13.6)	
**Recurrent stroke 3 months post‐stroke**												
No	28 (90.3)	26 (100)	60 (90.9)	.295*	62 (96.9)	18 (81.8)	31 (91.2)	.047*	69 (94.5)	19 (86.4)	20 (90.9)	.475*
Yes	3 (9.7)	0 (0.0)	6 (9.1)		2 (3.1)	4 (18.2)	3 (8.8)		4 (5.5)	3 (13.6)	2 (9.1)	
**Recurrent stroke 6 months post‐stroke**												
No					63 (98.4)	22 (100)	29 (85.3)	.023*	71 (97.3)	22 (100)	19 (86.4)	.114*
Yes					1 (1.6)	0 (0.0)	5 (14.7)		2 (2.7)	0 (0.0)	3 (13.6)	
**Recurrent stroke 12 months post‐stroke**												
No									73 (100)	21 (95.5)	18 (81.8)	.002*
Yes									0 (0.0)	1 (4.5)	4 (18.2)	
**Fatigue 3 months post‐stroke**												
No (FSS < 4)	7 (22.6)	1 (3.8)	0 (0.0)	< .001*	8 (12.5)	0 (0.0)	0 (0.0)	.026*	8 (11.0)	0 (0.0)	0 (0.0)	.109*
Yes (FSS ≥ 4)	24 (77.4)	25 (96.2)	66 (100)		56 (87.5)	22 (100)	34 (100)		65 (89.0)	22 (100)	22 (100)	
**Fatigue 6 months post‐stroke**												
No (FSS < 4)					17 (26.6)	2 (9.1)	1 (2.9)	**.007**	19 (26.0)	1 (4.5)	0 (0.0)	.002*
Yes (FSS ≥ 4)					47 (73.4)	20 (90.9)	33 (97.1)		54 (74.0)	21 (95.5)	22 (100)	
**Fatigue 12 months post‐stroke**												
No (FSS < 4)									47 (64.4)	3 (13.6)	3 (15.0)	**< .001**
Yes (FSS ≥ 4)									26 (35.6)	19 (86.4)	17 (85.0)	
**Pain 3 months post‐stroke**												
VAS ≤ 3	21 (67.7)	7 (26.9)	19 (29.2)	**< .001**	33 (51.6)	7 (31.8)	7 (20.6)	**.008**	35 (47.9)	8 (36.4)	3 (13.6)	**.015**
VAS ≥ 4	10 (32.3)	19 (73.1)	46 (70.8)		31 (48.4)	15 (68.2)	27 (79.4)		38 (52.1)	14 (63.6)	19 (86.4)	
**Pain 6 months post‐stroke**												
VAS ≤ 3					42 (65.6)	9 (40.9)	10 (31.3)	**.003**	46 (63.0)	8 (36.4)	6 (28.6)	**.006**
VAS ≥ 4					22 (34.4)	13 (59.1)	22 (68.8)		27 (37.0)	14 (63.6)	15 (71.4)	
**Pain 12 months post‐stroke**												
VAS ≤ 3									60 (83.3)	12 (54.5)	11 (55.0)	**.004**
VAS ≥ 4									12 (16.7)	10 (45.5)	9 (45.0)	
**Central pain 3 months post‐stroke**												
DN4 < 4	26 (83.9)	19 (73.1)	47 (72.3)	.447	54 (84.4)	14 (63.6)	22 (66.7)	.055	62 (84.9)	13 (59.1)	13 (61.9)	**.012**
DN4 ≥ 4	5 (16.1)	7 (26.9)	18 (27.7)		10 (15.6)	8 (36.4)	11 (33.3)		11 (15.1)	9 (40.9)	8 (38.1)	
**Central pain 12 months post‐stroke**												
DN4 < 4									72 (98.6)	17 (77.3)	16 (80.0)	.001*
DN4 ≥ 4									1 (1.4)	5 (22.7)	4 (20.0)	
**Shoulder pain 3 months post‐stroke**												
No	24 (80.0)	18 (69.2)	36 (54.5)	**.045**	47 (74.6)	16 (72.7)	14 (41.2)	**.003**	51 (69.9)	17 (77.3)	9 (40.9)	**.019**
Yes	6 (20.0)	8 (30.8)	30 (45.5)		16 (25.4)	6 (27.3)	20 (58.8)		22 (30.1)	5 (22.7)	13 (59.1)	
**Shoulder pain 6 months post‐stroke**												
No					49 (77.8)	15 (68.2)	16 (47.1)	**.009**	53 (72.6)	16 (72.7)	11 (50.0)	.121
Yes					14 (22.2)	7 (31.8)	18 (52.9)		20 (27.4)	6 (27.3)	11 (50.0)	
**Shoulder pain 12 months post‐stroke**												
No									66 (90.4)	20 (90.9)	14 (63.6)	.013*
Yes									7 (9.6)	2 (9.1)	8 (36.4)	
**Muscle spasticity 3 months post‐stroke**												
MAS < 3	28 (90.3)	26 (100)	44 (66.7)	**< .001**	61 (95.3)	18 (81.8)	16 (47.1)	**< .001**	66 (90.4)	18 (81.8)	9 (40.9)	< .001*
MAS ≥ 3	3 (9.7)	0 (0.0)	22 (33.3)		3 (4.7)	4 (18.2)	18 (52.9)		7 (9.6)	4 (18.2)	13 (59.1)	
**Muscle spasticity 6 months post‐stroke**												
MAS < 3					62 (96.9)	20 (90.9)	17 (50.0)	**< .001**	68 (93.2)	20 (90.9)	9 (40.9)	< .001*
MAS ≥ 3					2 (3.1)	2 (9.1)	17 (50.0)		5 (6.8)	2 (9.1)	13 (59.1)	
**Muscle spasticity 12 months post‐stroke**												
MAS < 3									72 (98.6)	19 (90.5)	11 (52.4)	< .001*
MAS ≥ 3									1 (1.4)	2 (9.5)	10 (47.6)	
**Subluxation of shoulder at 3 month post‐stroke**												
No	31 (100)	24 (92.3)	58 (89.2)	.167*	63 (98.4)	19 (86.4)	28 (84.8)	.012*	70 (95.9)	19 (86.4)	18 (85.7)	.108*
Yes	0 (0.0)	2 (7.7)	7 (10.8)		1 (1.6)	3 (13.6)	5 (15.2)		3 (4.1)	3 (13.6)	3 (14.3)	
**Subluxation of shoulder at 6 month post‐stroke**												
No					64 (100)	20 (90.9)	33 (100)	.033*	73 (100)	20 (90.9)	21 (100)	.066*
Yes					0 (0.0)	2 (9.1)	0 (0.0)		0 (0.0)	2 (9.1)	0 (0.0)	
**Joint contractures 3 months post‐stroke**												
No	27 (87.1)	17 (65.4)	32 (50.0)	**.002**	49 (76.6)	12 (57.1)	14 (42.4)	**.003**	54 (74.0)	10 (47.6)	9 (42.9)	**.008**
Yes	4 (12.9)	9 (34.6)	32 (50.0)		15 (23.4)	9 (42.9)	19 (57.6)		19 (26.0)	11 (52.4)	12 (57.1)	
**Joint contractures 6 months post‐stroke**												
No					53 (82.8)	14 (66.7)	13 (39.4)	**< .001**	57 (78.1)	12 (57.1)	10 (47.6)	**.013**
Yes					11 (17.2)	7 (33.3)	20 (60.6)		16 (21.9)	9 (42.9)	11 (52.4)	
**Joint contractures 12 months post‐stroke**												
No									67 (91.8)	16 (72.7)	12 (60.0)	.001*
Yes									6 (8.2)	6 (27.3)	8 (40.0)	
**Pressure ulcers 3 months post‐stroke**												
No (PU = 0)	28 (90.3)	19 (73.1)	35 (53.0)	**.001**	56 (87.5)	14 (63.6)	12 (35.3)	**< .001**	60 (82.2)	13 (59.1)	8 (36.4)	**< .001**
Yes (PU ≥ 1)	3 (2.4)	7 (26.9)	31 (47.0)		8 (12.5)	8 (36.4)	22 (64.7)		13 (17.8)	9 (40.9)	14 (63.6)	
**Pressure ulcers 6 months post‐stroke**												
No (PU = 0)					59 (92.2)	17 (77.3)	12 (36.4)	**< .001**	64 (87.7)	16 (72.7)	7 (31.8)	**< .001**
Yes (PU ≥ 1)					5 (7.8)	5 (22.7)	21 (63.6)		9 (12.3)	6 (27.3)	15 (68.2)	
**Pressure ulcers 12 months post‐stroke**												
No (PU = 0)									71 (97.3)	19 (86.4)	9 (40.9)	< .001*
Yes (PU ≥ 1)									2 (2.7)	3 (13.6)	13 (59.1)	
**Confirmed pneumonia 3 month post‐stroke**												
No	30 (96.8)	22 (84.6)	47 (72.3)	**.014**	60 (93.8)	18 (81.8)	19 (57.6)	**< .001**	65 (89.0)	17 (77.3)	14 (66.7)	.046*
Yes	1 (3.2)	4 (15.4)	18 (27.7)		4 (6.3)	4 (18.2)	14 (42.4)		8 (11.0)	5 (22.7)	7 (33.3)	
**Confirmed pneumonia 6 month post‐stroke**												
No					63 (98.4)	20 (90.9)	25 (75.8)	.001*	72 (98.6)	21 (95.5)	14 (66.7)	< .001*
Yes					1 (1.6)	2 (9.1)	8 (24.2)		1 (1.4)	1 (4.5)	7 (33.3)	
**Confirmed DVT 3 month post‐stroke**												
No	30 (96.8)	25 (96.2)	55 (83.3)	.084*	61 (95.3)	21 (95.5)	26 (76.5)	.012*	69 (94.5)	19 (86.4)	17 (77.3)	.048*
Yes	1 (3.2)	1 (3.8)	11 (16.7)		3 (4.7)	1 (4.5)	8 (23.5)		4 (5.5)	3 (13.6)	5 (22.7)	

*Note*: The results of all tables are presented in terms of frequencies and percentages and in the form of means and standard deviations. They are presented according to the mild (18 ≤ MMSE ≤ 23) and severe cognitive impairment (MMSE ≤ 17) occurring at 3, 6, and 12 months post‐stroke. The Chi‐square test was used to verify the association between the independent categorical variables and mild and severe cognitive sequelae.

Abbreviations: HADS_A, Hospital Anxiety and Depression_Anxiety; HADS_D, Hospital Anxiety and Depression_Depression; FSS, Fatigue Severity Scale; VAS, Visual Analogue Scale; DN4, *Douleur Neuropathique* 4 questionnaire; MAS, Modified Ashworth Scale; PU, pressure ulcers; DVT, deep vein thrombosis PSCI, Post‐Stroke Cognitive Impairment.

* Fisher's exact test was used in the event of an expected value *n* < 5. One way ANOVA test, Bonferroni method, was used to verify the association between means and mild and severe cognitive sequelae.

Their mean MMSE scores were 13.08 (± 6.52) and 25.84 (± 1.49), respectively (*p* < .001) at 3 months. They became 13.80 (± 6.92) and 26.69 (± 1.59) at 6 months versus 14.77 (± 6.72) and 28.37 (± 1.93) at 12 months, respectively (*p* < .001).

Factors that were significantly associated with severe cognitive function decline (MMSE ≤ 17) in the three time series post‐stroke were the atrial fibrillation (AF) (27% to 46%) (*p* ≤ .010), sedentary lifestyle ≥12 h/day (55% to 77%) (*p* < .001), NIHSS ≥21 (30% to 60%) (*p* < .001), mRS ≥3 (85% to 95%) (*p* < .001), and low scores of PCS and MCS of the QoL (<30) (*p* < .05).

In addition to the above, we also found some associations with various complications post‐stroke as follows: HADS_A ≥ 11 (35% to 60%) (*p* ≤ .022), HADS_D ≥ 11 (80% to 95%) (*p* < .001), physical disorders as FSS ≥ 4 (85% to 100%) (*p* ≤ .026), VAS ≥ 4 (45% to 86%) (*p* ≤ .015), MAS ≥ 3 (33% to 59%) (*p* < .001), joint contractures (50% to 60%) (*p* ≤ .013), falls at 3 and 6 months post‐stroke (35% to 60%) (*p* ≤ .006), pressure ulcers (47% to 68%) (*p* ≤ .001), and pneumonia at 3 months post‐stroke (28% to 42%) (*p* ≤ .046).

On the other hand, the relation with other determinants varied across these three time points.

At 3 months, a significant association was found between severe cognitive impairment (MMSE ≤ 17) and old age (*p* = .008) (81.8% of subjects were aged ≥ 65 years old) and the presence of a caregiver (47%) (*p* = .002). In addition, a mean duration of hospital stay of > 9 days was associated with mild to severe cognitive impairment (*p* < .001). Adversely, employment post‐stroke (45.2%) (*p =* .001) showed better cognitive function (MMSE >24).

At 6 months, falls (35.3%) (*p* = .003), epileptic seizures (17.6%) (*p* = .001), DVT at 3 months post‐stroke (23.5%) (*p* = .012), pneumonia that occurred at 6 months (24.2%) (*p* = .001), and recurrent stroke at 3 and 6 months post‐stroke (9% to 15%) (*p* ≤ .047), all affected the cognitive function of the recruited subjects resulting in severe damage (MMSE ≤ 17). Shoulder subluxation at 3 months (15.2%) (*p* = .012) was associated with MMSE ≤ 17, but its occurrence at 6 months (9.1%) was associated with 18 ≤ MMSE ≤ 23 (*p* = .033). However, high level of education (secondary or university education) (48.4%) (*p =* .049) presented a protective factor for the cognitive function (MMSE > 24) at 6 months.

At 12‐month follow‐up, age ≥ 65 years old (90.9%) (*p* = .028), having a left hemisphere stroke (45.5%) (*p* = .006), an ICU stay at stroke occurrence (59.1%) (*p* = 0.011), evidence of recurrent stroke at 12 months (18.2%) (*p* = 0.002), falls at 6 months post‐stroke (41%) (*p* = .006), shoulder pain at 12 months post‐stroke (36.4%) (*p* = .013), pneumonia at 6 months post‐stroke (33.3%) (*p* < .001), and DVT at 3 months post‐stroke (22.7%) (*p* = .048) showed a significant association with MMSE ≤ 17. Moreover, subjects suffering from neuropathic pain (DN4 ≥ 4) at 3 and 12 months presented with mild‐to‐severe cognitive impairment (20% to 40%) (*p* ≤ .012), whereas high educational level (45.2%) (*p* = .005) and use of antidiabetic treatment post‐stroke (55%) (*p* = .007) were associated with normal cognitive function (MMSE > 24).

Multivariable analysis using a multinomial logistic regression model was performed to identify the factors that were independently associated with cognitive impairment at 3, 6, and 12 months after stroke (shown in Table 5). Our data have shown an important independent contribution of different factors to the rate of mild or severe PSCI across the three periods.

**TABLE 5:** Independent determinants of post‐stroke cognitive impairment using multinomial logistic regression analysis

	Adjusted OR [95% CI]	*p*‐Value
**Factors associated with PSCI at 3 months** ^a^		
**Mild PSCI (18 ≤MMSE ≤23)**		
Employment post‐stroke	0.310 [0.113–0.853]	.023
HADS_A ≥ 11 at 3 month post‐stroke	2.698 [1.045–6.968]	.040
**Severe PSCI (MMSE ≤ 17)**		
Age	2.371 [1.191–4.720]	.014
Sedentary behavior (≥ 12 h/day)	3.062 [1.097–8.549]	.033
PCS score of QoL at 3 months	0.795 [0.704–0.897]	< .001
HADS_D ≥ 11 at 3 months post‐stroke	2.536 [1.004–6.403]	.049
**Factors associated with PSCI at 6 months** ^b^		
**Mild PSCI (18 ≤ MMSE ≤ 23)**		
Age	3.259 [1.482–7.166]	.003
Secondary or university education	0.059 [0.008–0.458]	.007
**Severe PSCI (MMSE ≤ 17)**		
Age	3.059 [1.442–6.487]	.004
NIHSS ≥ 21 at 3 months post‐stroke	3.231 [1.335–7.820]	.009
**Associated factors with PSCI at 12 months** ^c^		
**Mild PSCI (18 ≤ MMSE ≤ 23)**		
Gender, Female	0.087 [0.010–0.755]	.027
Age	2.378 [1.164–4.856]	.017
Secondary or university education	0.115 [0.021–0.625]	.012
PCS score of QoL at 12 months	0.833 [0.719–0.965]	.015
Use of anti‐diabetic medication post‐stroke	0.166 [0.039–0.712]	.016
HADS_D ≥ 11 at 3 months post‐stroke	3.490 [1.256–9.695]	.017
**Severe PSCI (MMSE ≤ 17)**		
Age	2.186 [1.037–4.608]	.040
Secondary or university education	0.159 [0.027–0.951]	.044
Stroke location, left hemisphere	2.710 [1.328–5.528]	.006
NIHSS score at 3 months post‐stroke	3.375 [1.483–7.678]	.004
PCS score of QoL at 12 months	0.808 [0.684–0.954]	.012
Use of anti‐diabetic medication post‐stroke	0.182 [0.037–0.894]	.036

Abbreviations: AOR, adjusted odds ratio; CI, confidence interval; PSCI, Post‐stroke Cognitive Impairment; MMSE, Mini‐Mental State Examination; HADS_A, Hospital Anxiety and Depression Scale_Anxiety score; PCS of QoL, Physical Component Summary of the Short Form Health survey (SF12) for evaluation of the quality of life; HADS_D, Hospital Anxiety and Depression Scale_Depression score; NIHSS, National Institutes of Health Stroke Scale.

^a^ Classification table: Global percentage 74.6%, Model fit test < .001, Nagelkerke R‐Square 0.630, Goodness of fit test 1.000.

^b^ Classification table: Global percentage 78.3%, Model fit test < .001, Nagelkerke R‐Square 0.666, Goodness of fit test 1.000.

^c^ Classification table: Global percentage 82.5%, Model fit test < .001, Nagelkerke R‐Square 0.669, Goodness of fit test 1.000.

All of these multivariate analyses included all variables and confounding factors that had a value of *p* ≤ .05 in the univariate analysis. The method of selection of the variables which has been chosen here is the backward stepwise method. The reference category is the absence of PSCI (MMSE ≥ 24).

At 3 months post‐stroke, anxiety was positively associated with 18 ≤ MMSE ≤ 23 (AOR = 2.698, 95% CI = [1.045 – 6.968], *p* = 0.040). Whereas, old age, sedentary lifestyle (≥ 12 h/day), and depression 3‐month post‐stroke increased two to three times the risk of severe cognitive impairment (MMSE ≤ 17) (AOR = 2.371, 95% CI = [1.191–4.720], *p* = .014; AOR = 3.062, 95% CI = [1.097–8.549], *p* = .033; AOR = 2.536, 95% CI = [1.004–6.403], *p* = .049, respectively). In contrast, subjects who resumed work post‐stroke and had higher PCS scores of QoL were less likely to develop PSCI (AOR = 0.310, 95% CI = [0.113–0.853], *p* = .023; AOR = 0.795, 95%CI = [0.704–0.897], *p* < .001).

At 6 months, old age (AOR = 3.059, 95% CI = [1.442–6.487], *p* = .004) and severe stroke (NIHSS ≥ 21) (AOR = 3.231, 95% CI = [1.335–7.820], *p* = .009) affected severely the cognitive function (MMSE ≤ 17), whereas high educational level presented as a protective factor for the cognitive function (AOR = 0.059, 95% CI = [0.008–0.458], *p* = .007).

At the 12‐month follow‐up, old age, HADS_D ≥ 11, high NIHSS scores, continued to exhibit a significant contribution to induce an impairment of the cognitive function (AOR = 2.186, 95% CI = [1.037–4.608], *p =* .040; AOR = 3.490, 95% CI = [1.256–9.695], *p =* .017; AOR = 3.375, 95% CI = [1.483–7.688], *p =* .004, respectively). Moreover, we noted that the left hemisphere stroke increased approximately threefold the risk of late cognitive decline (AOR = 2.710, 95% CI = [1.328–5.528], *p* = .006).

On the other hand, females were 91% less likely to develop MCI at 12 months post‐stroke (AOR = 0.087, 95%CI = [0.010–0.755], *p* = .027), and the subjects who were on anti‐diabetic treatment post‐stroke had 82% lower risk of 12‐month mild to severe cognitive impairment (AOR = 0.182, 95% CI = [0.037–0.894], *p* = .036). Similarly, high educational level and higher PCS scores of QoL stayed having a significant association with higher scores of MMSE at 12 months post‐stroke (AOR = 0.159, 95% CI = [0.027–0.951], *p* = .044; AOR = 0.808, 95% CI = [0.684–0.954], *p* = .012, respectively).

## 4 DISCUSSIONS

To the best of our knowledge, this is the first study of its kind in Lebanon. The main objective was to calculate the MMSE scores among stroke survivors, to evaluate the different cognitive domains and to determine the proportion and the predictors of PSCI at three time points post first‐ever stroke: 3, 6, and 12 months. Overall, PSCI was very high in the first‐ever stroke survivors ranging between 74.8% in the early stage and 37.6% in the late stage. Subjects with a cognitive impairment at an acute phase had very low MMSE scores (< 17) that were associated with poorer functional outcome and QoL. Age, low educational level, high NIHSS scores, low PCS of QoL, sedentary lifestyle, and high HADS_A and HADS_D scores were the main independent predictors of the deficit in MMSE scores among Lebanese stroke survivors. A significant substantial 37% improvement of the cognitive function was seen at 1 year post‐stroke.

Compared to different eastern and western countries, PSCI rate at acute phase in Lebanon is one of the highest rates, similar to the rates in Shanghai, China (88.1%) (Li et al., 2020), and South Korea (69.8%) (Yu et al., 2013), but greater than the rates obtained in USA, France, Britain, Australia, Sweden, India, Norway, Singapore, Nigeria, and Egypt that range between 20% and 50% (Akinyemi et al., 2014; Essmat et al., 2021; Sun et al., 2014). This variability might be explained by the difference in countries, race, and the used diagnostic criteria, in addition to different factors reported in the present study.

Various factors were identified as the determinants of PSCI over 1 year of follow‐up among Lebanese survivors.

Age was the main predictor across the three time points. PSCI decreases exponentially as age increases (Levine et al., 2018; Sun et al., 2014), which can be explained in biological terms by the deposition of β‐amyloid, one of the pathological hallmarks of Alzheimer's disease (Doré et al., 2013).

Interestingly, males were more likely to be affected than females. This observation was statistically significant in the late phase (1‐year post‐stroke) with mild cognition decline (18 ≤ MMSE ≤ 23). This was found inconsistent with the results of most of the previous studies (Au et al., 2017; Jia et al., 2020; Levine et al., 2018).This could be explained by the fact that males in our study have had more comorbidities than females and were more exposed to psychological complications. In addition to these possible reasons that were discussed by Wang and collaborators in 2020 (Wang et al., 2020), some studies reported that gender difference varies according to the type of dementia, with higher rate of Alzheimer's disease in females and more vascular dementia in males (Au et al., 2017; Gannon et al., 2019).

Moreover, previous studies have shown that high educational level and employment post‐stroke are associated with better cognitive performances (Hatem et al., 2016; Kemp et al., 2019; Levine et al., 2018; Mahon et al., 2017; Sun et al., 2014). People with higher reserve can endure more neurological problems and can maintain brain function for longer periods of time than people with lower reserve. They have a more favorable and healthier lifestyle, better compliance to treatment and better access to healthcare leading to less cognitive decline and dementia (Srithumsuk et al., 2020). However, in the present study, illiteracy and lower educational level were remarkable among participants as well as unemployment post‐stroke, consequently affecting their cognitive function.

Our results showed that the odds of severe PSCI increased three times when sitting hours increased to ≥ 12 h/day, which was highlighted also by a study conducted among five population cohorts from Greece, Australia, USA, Japan, and Singapore in 2020 (Maasakkers et al., 2020). Stroke survivors tend to be less active, spend more time sedentary compared to stroke‐free people (Viktorisson et al., 2021). They become less energetic, less concentrated, less motivated, more depressed, more anxious, and more likely to isolate themselves without any accomplishment. Therefore, regular physical activity of at least 30 min of moderate intensity one to three times per week during stroke rehabilitation phase is highly recommended as part of the secondary prevention by the American Heart Association (2021).

Furthermore, AF was found to be a significant risk factor for mild and severe PSCI at early and late stage; in accordance with the literature, AF may result in downstream cerebral hypoperfusion, heightened inflammatory responses, silent ischemia, reduced brain volumes, and cerebral microbleeds, all of which have been implicated in PSCI (Morsund et al., 2019; Siennicki‐Lantz et al., 2008); however, this effect was lost when considering confounding risk factors in our study.

Inversely, the use of antidiabetic medication in our population was shown to be an independent protective factor against PSCI at 12 months post‐stroke similarly to the study of Swardfager and MacIntosh (2017). Therefore, we must highlight the most important approaches in the prevention of cognitive sequelae, including an optimal glycemic control and a healthier lifestyle intervention such as a proper diet and physical activity as mentioned earlier in the paper.

Pendlebury and Rothwell reviewed 73 cohort studies of post‐stroke dementia in a total of 7511 patients and they shed light on the importance of the stroke burden itself (stroke severity, location of the stroke: left hemisphere, volume of the infarct) in the cause of PSCI (Pendlebury & Rothwell, 2009). This is comparable to our findings. The involvement of the left hemisphere was shown to be an independent predictor of severe cognitive impairment at 1 year post‐stroke, increasing threefold the risk of PSCI. Regarding the stroke subtypes, MCI was found slightly higher in lacunar stroke (SVO) compared to non‐lacunar stroke (LAA) but was not statistically significant, similarly to the systematic review and meta‐analyses of Makin et al. (S. D. Makin et al., 2018; S. D. J. Makin et al., 2013). SVO is the most common vascular cause of cognitive impairment affecting the brain diffusely. In addition, the duration of hospital stay > 9 days at the stroke occurrence was associated with decreasing scores of MMSE in the following 3 to 6 months. Prolonged hospitalization worsens the situation of patients, exposing them to serious complications and more sequelae (Mathews et al., 2014).

In the rehabilitation phase, survivors with PSCI are more likely to be dependent in ADL, having high stroke severity and poorer mental and physical QoL (Mohd Zulkifly et al., 2016). Similarly, in the present study, increasing NIHSS scores, HADS scores, and mRS scores and decreasing PCS and MCS and SF‐12 of QoL at 3, 6, and 12 months were positively associated with lower MMSE scores over time. After adjusting for age, gender, educational level, and severity of stroke, anxiety and depression post‐stroke (HADS_A and HADS_D scores ≥ 11), in addition to the lower PCS of QoL, remained as independent strong predictors of PSCI across the three time periods of follow‐up. Motor deficit after stroke, including falls, general pain, neuropathic pain at 3 and 12 month, fatigue, shoulder subluxation, joint contractures, muscle spasticity, DVT, and pressure ulcers were contributing to the reduction in MMSE scores, similarly to numerous international studies (Graber et al., 2019; Harrison & Field, 2015; Lui & Nguyen, 2018; Renjen et al., 2015; Segev‐Jacubovski et al., 2011). Survivors usually experience sleep disturbances, low motivation, low self‐esteem, mood changes, chronic stress, and worries about their future due to disabilities. All these physical and emotional limitations affect their performance in executive function, memory, speed, and motor processing (Quattropani et al., 2018; Mohd Zulkifly et al., 2016). Hence, an emergency rehabilitation program for patients and their families is essential to enable them to achieve their highest possible level of independence. There is a crucial early need to focus on controlling motor movement and psychological factors in the different stages of stroke.

Last, we found that pneumonia post‐stroke induced short‐term and long‐term severe cognitive impairment. Emerging evidence suggests that immune responses are implicated in long‐term cognitive decline and dementia after stroke (Elkind et al., 2020; Wille‐Jørgensen et al., 2005). Autoantibodies against myelin basic protein are associated with increased cognitive decline in the first year after stroke (Becker et al., 2016).

Thus, concurrent impairments should be recognized both in a short‐ and long‐term perspective in order to identify and target the patients in need of prolonged rehabilitation to prevent further functional decline.

### 4.1 Strengths and Limitations

A number of limitations require consideration. First, the main limitation was the small sample size recruited following the unique study considering a low prevalence of stroke in Lebanon (Jurjus et al., 2009) of 3.9% according to other countries. Second, we failed to compare baseline scores of NIHSS, mRS, and MMSE with the three time series scores because of lack of assessment tools in the participating hospitals. Third, these latter were limited to the regions of Beirut and Mount Lebanon, regardless of the fact that subjects came from all governorates; this affected the generalizability of our results. Fourth, despite the results which were statistically significant, there was a loss to follow up due to the number of deaths at 3 months, the fact that we could not assess their cognitive function that was highly suspected to be impaired; therefore, we believe that the cognitive function is underestimated in our study. Fifth, we should mention that illiteracy in the present study (38% of the participants) was an important confounder and it is well known that illiteracy tends to affect cognitive functioning.

Nevertheless, our study was a multicenter longitudinal prospective study and one of the few such studies carried out in Lebanon which used international standardized, validated, and reliable measuring instruments. We have tried to avoid information bias by using the Arabic versions of these instruments. These instruments were performed by highly qualified and well‐trained investigators through a face‐to‐face interview with the subjects so the degree of bias usually resulting from self‐completed questionnaires due to misunderstanding of the questions was declined. On the one hand, we have employed measures to tap different aspects of post‐stroke sequelae. This is, indeed, the novelty of this study. On the other hand, since the subjects were followed for 12 months, some of the measures were repeated, and it is unclear whether there was an effect on learning (Wesnes & Pincock, 2002). Similarly, another confounding factor should be considered, the phenomenon known as spontaneous recovery in people who have sustained neurological events (Cramer, 2008). Lastly, PSCI is a multifactorial disorder, so we did not miss and relatively considered the majority of significant potential explanatory factors.

Hence, conclusions should be confirmed in a larger cohort. Our findings may be useful to draw hypotheses on stroke burden and its heavy physical and mental implications for further analyses. Future studies should take into account all the weak points and a larger sample size across Lebanon must be considered to confirm our findings.

## 5 CONCLUSIONS

PSCI is a major cause of handicap, morbidity, and mortality. We concluded that PSCI rate at acute phase in Lebanon is one of the highest rates worldwide. A substantial improvement of the cognitive function was highlighted at 1 year post initial stroke. Various factors were identified as major determinants among Lebanese survivors. We must shed light on the early phase post‐stroke, which is the most critical and sensitive phase affecting the short‐ and long‐term cognitive functioning post‐stroke. Stroke patients should be closely and regularly monitored in the rehabilitation phase, especially in the early phase. Appropriate evaluation of cognitive function, identification of the risk factors, and providing more comprehensive assessment to stroke survivors from the beginning would be useful in the post‐stroke phase. A standardized protocol and rehabilitation program to cope with the burden of stroke should be implemented to best address the management of the risk factors, including broad neuropsychological evaluation besides screening measures to improve the functional outcome, the cognitive function, and to prevent associated consequences. Finally, there is a need to increase public awareness. A valuable coordination, collaboration among healthcare professionals and a solid support from the families and caregivers can contribute to the fast and smooth recovery of stroke survivors.

## AUTHOR CONTRIBUTIONS

Hassan Hosseini, Pascale Salameh, and Celina Boutros contributed to the conception and design of the study. Celina Boutros, Walaa Khazaal, and Maram Taliani organized the database. Celina Boutros performed the statistical analysis, and wrote the first draft and the sections of the manuscript. All authors contributed to manuscript revision and read and approved the submitted version.

## CONFLICT OF INTEREST

The authors have no conflict of interest to declare.

## FUNDING INFORMATION

The authors declare that this study received funding from Association Robert Debré pour la Recherche Médicale (ARDRM). The funder was not involved in the study design,collection, analysis, interpretation of data, the writing of this article or the decision to submit it for publication.

### PEER REVIEW

The peer review history for this article is available at: https://publons.com/publon/10.1002/brb3.2837

## Supporting information

Supplementary Information

Figure 1S. The severity of the stroke measured by the National Institutes of Health Stroke Scale (NIHSS). It is divided into 5 levels: 0: no stroke, 1–4: minor stroke, 5–15: moderate stroke, 15–20: moderate to severe stroke, 21–42: severe stroke.

Figure 2S. The degree of disability measured by the modified Rankin Scale (mRS). It is divided into 7 levels as follows: 0: no symptoms; 1: no significant disability despite symptoms; able to perform all the duties and usual activities; 2: low incapacity; unable to accomplish many previous things, but capable of taking care of its own affairs without assistance; 3: inability to moderate; needing help, but able to walk without assistance; 4: moderately serious disability; unable to walk without assistance and unable to meet physical needs without assistance; 5: serious disability; bedridden, incontinent and demanding necessary attention and constant nursing care; 6: death.

Figure 3S. The rates of cognitive impairment occurring after 3, 6, and 12‐month post‐stroke.

Figure 4S. The percentage of subjects with/without cognitive domain impairment according to MMSE across the 3 periods of follow‐up.

## Data Availability

Data are available upon request due to privacy and ethical restrictions.
