# Supplementary material for: Factors associated with cognitive impairment at 3, 6, and 12 months after the first stroke among Lebanese survivors
Source: Brain Behav. 2022 Dec 10;13(1):e2837. doi: 10.1002/brb3.2837 (PMC9847618; doi:10.1002/brb3.2837)
Supplement: Supplementary file 1 — Supplementary Information [file BRB3-13-e2837-s001.docx]

**Supplementary material**


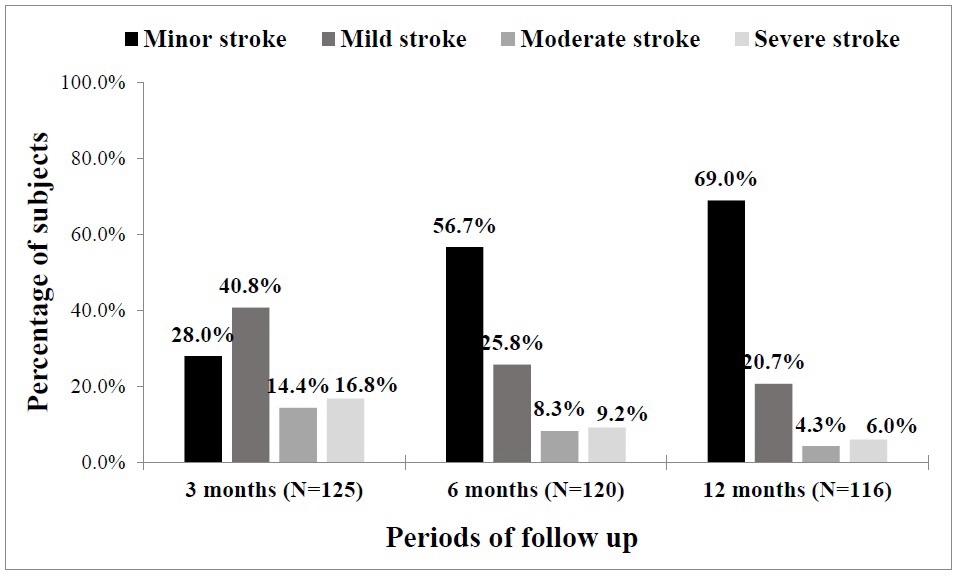
**Figure 1S. The severity of the stroke measured by the National Institutes of Health Stroke Scale (NIHSS).** It is divided into 5 levels: 0: No stroke, 1-4: minor stroke, 5-15: moderate stroke, 15-20: moderate to severe stroke, 21-42: severe stroke.

**
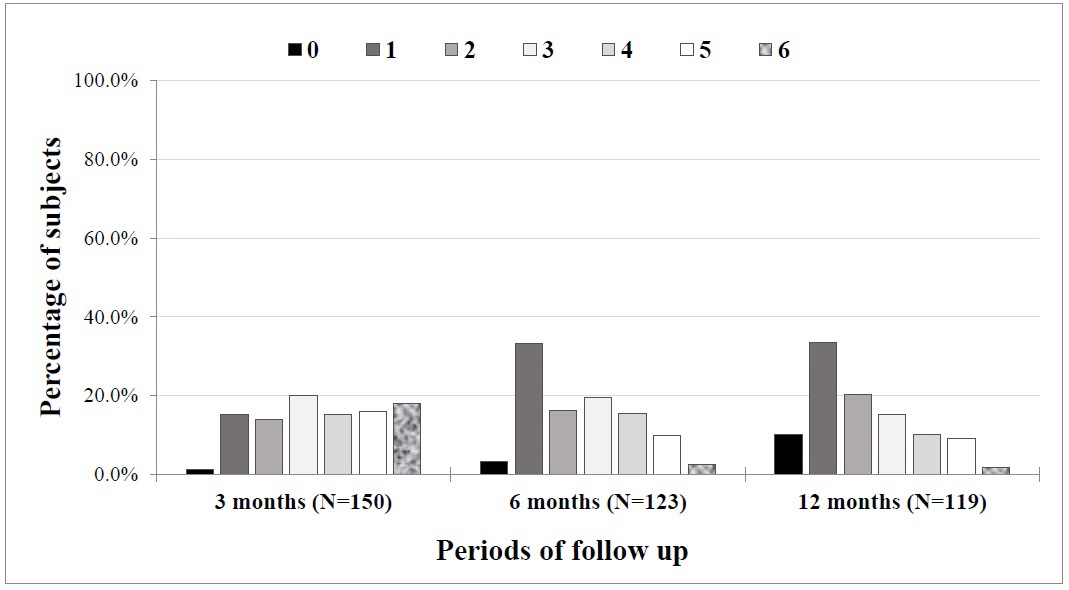
Figure 2S. The degree of disability measured by the modified Rankin Scale (mRS).** It is divided into 7 levels as follows: 0: No symptoms; 1: No significant disability despite symptoms; able to perform all the duties and usual activities; 2: Low incapacity; unable to accomplish many previous things, but capable of taking care of its own affairs without assistance; 3: inability to moderate; needing help, but able to walk without assistance; 4: Moderately serious disability; unable to walk without assistance and unable to meet physical needs without assistance; 5: Serious disability; bedridden, incontinent and demanding necessary attention and constant nursing care; 6: Death.

**Table 1S. The quality of life measured by the SF-12 (Short Form Health Survey).**

|  | **3 months** | **6 months** | **12 months** |
| --- | --- | --- | --- |
| **Short Form Health Survey (SF-12)** | **Mean (±SD)** | **Mean (±SD)** | **Mean (±SD)** |
| ***Physical component summary (PCS)*** |  |  |  |
| *General Health (GH)* | *4.35 (±0.80)* | *3.78 (±0.97)* | *3.42 (±1.17)* |
| *Physical Functioning (PF)* | *2.64 (±0.99)* | *3.22 (±1.29)* | *3.72 (±1.65)* |
| *Role limitations due to physical health (RP)* | *2.27 (±0.61)* | *2.50 (±0.81)* | *2.82 (±0.96)* |
| *Bodily Pain (BP)* | *3.66 (±1.26)* | *2.94 (±1.24)* | *2.37 (±1.34)* |
| ***Total PCS*** | ***28.96 (±7.31)*** | ***34.92 (±9.21)*** | ***39.49 (±11.30)*** |
| ***Mental component summary (MCS)*** |  |  |  |
| *Vitality (VT)* | *4.58 (±1.29)* | *4.15 (±1.37)* | *3.70 (±1.55)* |
| *Social Functioning (SF)* | *2.22 (±0.89)* | *2.49 (±1.10)* | *3.03 (±1.43)* |
| *Role limitations due to emotional health (RE)* | *2.32 (±0.66)* | *2.50 (±0.81)* | *2.84 (±0.96)* |
| *Mental health (MH)* | *7.25 (±1.11)* | *7.10 (±1.23)* | *7.02 (±1.50)* |
| ***Total MCS*** | ***32.65 (±9.41)*** | ***35.17(±10.44)*** | ***40.12 (±12.85)*** |

PCS: Physical Component Summary; MCS: Mental Component Summary; SD: Standard Deviation.


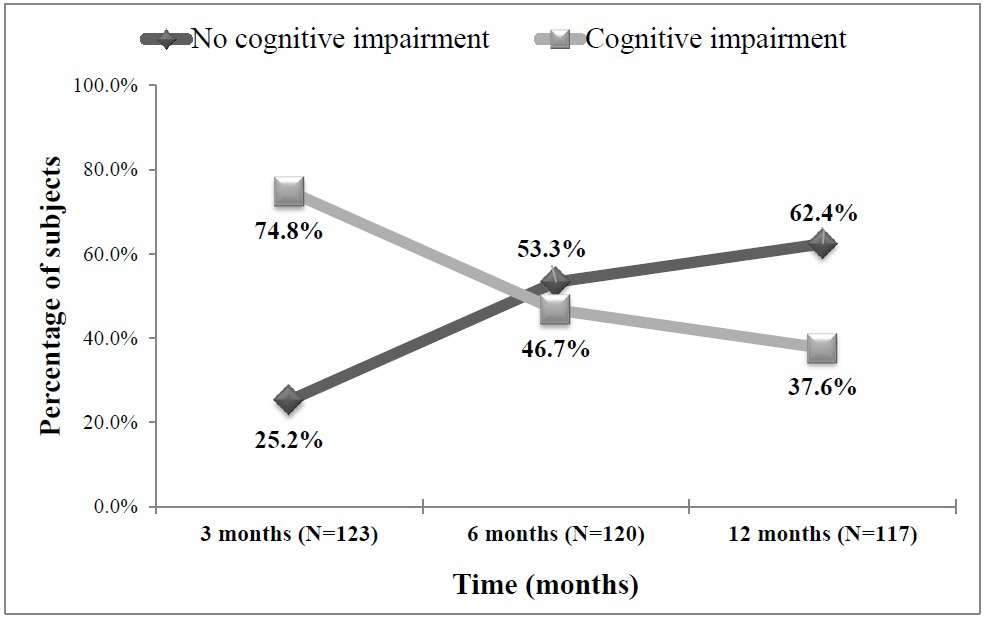


**Figure 3S. The rates of cognitive impairment occurring after 3, 6 and 12 month post-stroke.**


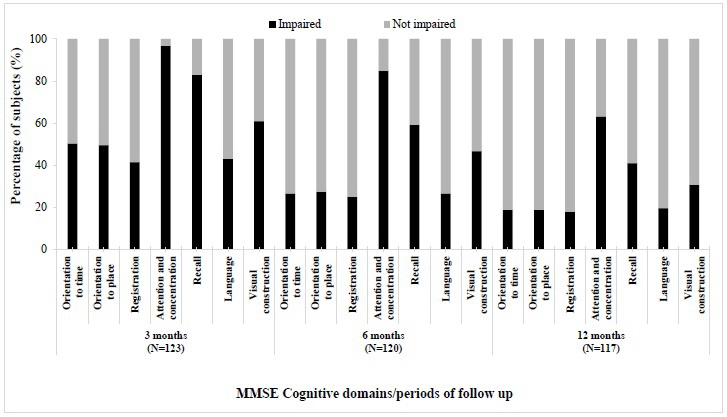


**Figure 4S. The percentage of subjects with/without cognitive domain impairment according to MMSE across the 3 periods of follow-up.**

**Table 2S. The association between pre-existing conditions and the post-stroke cognitive impairment among Lebanese stroke survivors.**

| **Pre-existing**  **conditions** | **3 months post stroke** | | | | **6 months post stroke** | | | | **12 months post stroke** | | | |
| --- | --- | --- | --- | --- | --- | --- | --- | --- | --- | --- | --- | --- |
|  | **No**  **PSCI**  **N (%)** | **Mild PSCI**  **N (%)** | **Severe PSCI**  **N (%)** | **p-value** | **No**  **PSCI**  **N (%)** | **Mild PSCI**  **N (%)** | **Severe PSCI**  **N (%)** | **p-value** | **No**  **PSCI**  **N (%)** | **Mild PSCI**  **N (%)** | **Severe PSCI**  **N (%)** | **p-value** |
| **Atrial Fibrillation** | |  |  |  |  |  |  |  |  |  |  |  |
| *No* | 30 (96.8) | 23 (88.5) | 48 (72.7) | **0.010** | 60 (93.8) | 15 (68.2) | 24 (70.6) | **0.002** | 67 (91.8) | 17 (77.3) | 12 (54.5) | **<0.001*** |
| *Yes* | 1 (3.2) | 3 (11.5) | 18 (27.3) |  | 4 (6.3) | 7 (31.8) | 10 (29.4) |  | 6 (8.2) | 5 (22.7) | 10 (45.5) |  |
| **Myocardial Infarction** | |  |  |  |  |  |  |  |  |  |  |  |
| *No* | 31 (100) | 24 (92.3) | 64 (97.0) | 0.245* | 62 (96.9) | 21 (95.5) | 33 (97.1) | 1.000* | 71 (97.3) | 21 (95.5) | 21 (95.5) | 0.631* |
| *Yes* | 0 (0.0) | 2 (7.7) | 2 (3.0) |  | 2 (3.1) | 1 (4.5) | 1 (2.9) |  | 2 (2.7) | 1 (4.5) | 1 (4.5) |  |
| **High blood pressure** | |  |  |  |  |  |  |  |  |  |  |  |
| *No* | 9 (29.0) | 3 (11.5) | 17 (25.8) | 0.249 | 14 (21.9) | 4 (18.2) | 10 (29.4) | 0.576 | 17 (23.3) | 5 (22.7) | 6 (27.3) | 0.919 |
| *Yes* | 22 (71.0) | 23 (88.5) | 49 (74.2) |  | 50 (78.1) | 18 (81.8) | 24 (70.6) |  | 56 (76.7) | 17 (77.3) | 16 (72.7) |  |
| **Other cardiovascular diseases** | | |  |  |  |  |  |  |  |  |  |  |
| *No* | 27 (87.1) | 23 (88.5) | 55 (83.3) | 0.837* | 52 (81.3) | 20 (90.9) | 32 (94.1) | 0.189* | 61 (83.6) | 20 (90.9) | 20 (90.9) | 0.588* |
| *Yes* | 4 (12.9) | 3 (11.5) | 11 (16.7) |  | 12 (18.8) | 2 (9.1) | 2 (5.9) |  | 12 (16.4) | 2 (9.1) | 2 (9.1) |  |
| **Diabetes mellitus** | |  |  |  |  |  |  |  |  |  |  |  |
| *No* | 15 (48.4) | 14 (53.8) | 44 (66.7) | 0.189 | 33 (51.6) | 16 (72.7) | 21 (61.8) | 0.197 | 35 (47.9) | 17 (77.3) | 16 (72.7) | 0.051 |
| *Yes* | 16 (51.6) | 12 (46.2) | 22 (33.3) |  | 31 (48.4) | 6 (27.3) | 13 (38.2) |  | 38 (52.1) | 5 (22.7) | 6 (27.3) |  |
| **Dyslipidemia** |  |  |  |  |  |  |  |  |  |  |  |  |
| *No* | 12 (38.7) | 13 (50.0) | 33 (50.0) | 0.553 | 29 (45.3) | 11 (50.0) | 16 (47.1) | 0.929 | 34 (46.6) | 11 (50.0) | 10 (45.5) | 0.948 |
| *Yes* | 19 (61.3) | 13 (50.0) | 33 (50.0) |  | 35 (54.7) | 11 (50.0) | 18 (52.9) |  | 39 (53.4) | 11 (50.0) | 12 (54.5) |  |
| **Family history of stroke** |  |  |  |  |  |  |  |  |  |  |  |  |
| *No* | 8 (44.4) | 8 (47.1) | 28 (58.3) | 0.517 | 25 (55.6) | 6 (46.2) | 11 (50.0) | 0.805 | 29 (54.7) | 6 (46.2) | 6 (46.2) | 0.774 |
| *Yes* | 10 (55.6) | 9 (52.9) | 20 (41.7) |  | 20 (44.4) | 7 (53.8) | 11 (50.0) |  | 24 (45.3) | 7 (53.8) | 7 (53.8) |  |

PSCI: Post-stroke cognitive impairment; N: Frequency; %: Percentage.

**Table 3S. The association between lifestyle and the post-stroke cognitive impairment among Lebanese stroke survivors.**

| **Lifestyle** | **3 months post stroke** | | | | **6 months post stroke** | | | | **12 months post stroke** | | | |
| --- | --- | --- | --- | --- | --- | --- | --- | --- | --- | --- | --- | --- |
|  | **No**  **PSCI**  **N (%)** | **Mild PSCI**  **N (%)** | **Severe PSCI**  **N (%)** | **p-value** | **No**  **PSCI**  **N (%)** | **Mild PSCI**  **N (%)** | **Severe PSCI**  **N (%)** | **p-value** | **No**  **PSCI**  **N (%)** | **Mild PSCI**  **N (%)** | **Severe PSCI**  **N (%)** | **p-value** |
| **Smoking status** | |  |  |  |  |  |  |  |  |  |  |  |
| *Non smoker* | 12 (38.7) | 6 (23.1) | 28 (42.4) | 0.261 | 18 (28.1) | 12 (54.5) | 15 (44.1) | 0.052 | 25 (34.2) | 11 (50.0) | 8 (36.4) | 0.523 |
| *Ex-smoker* | 7 (22.6) | 7 (26.9) | 20 (30.3) |  | 17 (26.6) | 4 (18.2) | 12 (35.3) |  | 19 (26.0) | 5 (22.7) | 8 (36.4) |  |
| *Current smoker* | 12 (38.7) | 13 (50.0) | 18 (27.3) |  | 29 (45.3) | 6 (27.3) | 7 (20.6) |  | 29 (39.7) | 6 (27.3) | 6 (27.3) |  |
| **BMI** |  |  |  |  |  |  |  |  |  |  |  |  |
| *Normal weight (BMI≤25)* | 11 (36.7) | 13 (50.0) | 28 (42.4) | 0.745* | 27 (42.9) | 9 (40.9) | 13 (38.2) | 0.548* | 30 (41.7) | 10 (45.5) | 8 (36.4) | 0.582* |
| *Overweight (26≤BMI≤30)* | 11 (36.7) | 10 (38.5) | 21 (31.8) |  | 23 (36.5) | 9 (40.9) | 10 (29.4) |  | 27 (37.5) | 7 (31.8) | 6 (27.3) |  |
| *Obesity (31≤BMI≤40)* | 7 (23.3) | 3 (11.5) | 16 (24.2) |  | 12 (19.0) | 3 (13.6) | 11 (32.4) |  | 14 (19.4) | 4 (18.2) | 8 (36.4) |  |
| *Morbid obesity (BMI≥41)* | 1 (3.3) | 0 (0.0) | 1 (1.5) |  | 1 (1.6) | 1 (4.5) | 0 (0.0) |  | 1 (1.4) | 1 (4.5) | 0 (0.0) |  |
| **Mediterranean diet** | |  |  |  |  |  |  |  |  |  |  |  |
| *No* | 4 (12.9) | 2 (7.7) | 14 (21.2) | 0.241 | 10 (15.6) | 4 (18.2) | 5 (14.7) | 0.939 | 9 (12.3) | 4 (19.0) | 5 (22.7) | 0.407* |
| *Yes* | 27 (87.1) | 24 (92.3) | 52 (78.8) |  | 54 (84.4) | 18 (81.8) | 29 (85.3) |  | 64 (87.7) | 18 (81.8) | 17 (77.3) |  |
| **Regular physical activity (≥ 30min/day)** | | |  |  |  |  |  |  |  |  |  |  |
| *No* | 25 (83.3) | 23 (88.5) | 58 (90.6) | 0.516* | 51 (83.6) | 22 (100) | 30 (88.2) | 0.110* | 58 (82.9) | 21 (100) | 20 (90.9) | 0.106* |
| *Yes* | 5 (16.7) | 3 (11.5) | 6 (9.4) |  | 10 (16.4) | 0 (0.0) | 4 (11.8) |  | 12 (17.1) | 0 (0.0) | 2 (9.1) |  |
| **Sedentary duration** | |  |  |  |  |  |  |  |  |  |  |  |
| *1 to 6 hours/day* | 20 (64.5) | 7 (28.0) | 7 (11.3) | **<0.001** | 32 (53.3) | 1 (4.8) | 1 (2.9) | **<0.001** | 31 (45.6) | 2 (9.1) | 0 (0.0) | **<0.001** |
| *7 to 11 hours/day* | 8 (25.8) | 12 (48.0) | 21 (33.9) |  | 20 (33.3) | 13 (61.9) | 8 (23.5) |  | 25 (36.8) | 11 (50.0) | 5 (22.7) |  |
| ≥ *12 hours/day* | 3 (9.7) | 6 (24.0) | 64 (54.8) |  | 8 (13.3) | 7 (33.3) | 25 (73.5) |  | 12 (17.6) | 9 (40.9) | 17 (77.3) |  |
| **Social support (SSRS)** |  |  |  |  |  |  |  |  |  |  |  |  |
| *low level (SSRS≤22)* | 6 (19.4) | 5 (19.2) | 23 (34.8) | 0.157 | 17 (26.6) | 5 (22.7) | 10 (29.4) | 0.858 | 17 (23.3) | 6 (27.3) | 9 (40.9) | 0.267 |
| *Moderate level (22≤SSRS≤44)* | 25 (80.6) | 21 (80.8) | 43 (65.2) |  | 47 (73.4) | 17 (77.3) | 24 (70.6) |  | 56 (76.7) | 16 (72.7) | 13 (59.1) |  |
| *High level (SSRS≥45)* | 0 (0.0) | 0 (0.0) | 0 (0.0) |  | 0 (0.0) | 0 (0.0) | 0 (0.0) |  | 0 (0.0) | 0 (0.0) | 0 (0.0) |  |

PSCI: Post-stroke cognitive impairment; N: Frequency; %: Percentage; BMI: Body Mass Index; SSRS: Social Support Rating Scale.

**Table 4S. The association between medications taken post stroke and the post-stroke cognitive impairment among Lebanese stroke survivors.**

| **Treatment post stroke** | **3 months post stroke** | | | | **6 months post stroke** | | | | **12 months post stroke** | | | |
| --- | --- | --- | --- | --- | --- | --- | --- | --- | --- | --- | --- | --- |
|  | **No**  **PSCI**  **N (%)** | **Mild PSCI**  **N (%)** | **Severe PSCI**  **N (%)** | **p-value** | **No**  **PSCI**  **N (%)** | **Mild PSCI**  **N (%)** | **Severe PSCI**  **N (%)** | **p-value** | **No**  **PSCI**  **N (%)** | **Mild PSCI**  **N (%)** | **Severe PSCI**  **N (%)** | **p-value** |
| **Lipid lowering drug** | |  |  |  |  |  |  |  |  |  |  |  |
| *No* | 9 (29.0) | 9 (34.6) | 17 (25.8) | 0.696 | 19 (29.7) | 5 (22.7) | 11 (32.4) | 0.735 | 24 (32.9) | 4 (18.2) | 6 (27.3) | 0.404 |
| *Yes* | 22 (71.0) | 17 (65.4) | 49 (74.2) |  | 45 (70.3) | 17 (77.3) | 23 (67.6) |  | 49 (67.1) | 18 (81.8) | 16 (72.7) |  |
| **Anti-hypertensive drug** | |  |  |  |  |  |  |  |  |  |  |  |
| *No* | 8 (25.8) | 3 (11.5) | 17 (25.8) | 0.307 | 13 (20.3) | 4 (18.2) | 10 (29.4) | 0.511 | 16 (21.9) | 5 (22.7) | 6 (27.3) | 0.872 |
| *Yes* | 23 (74.2) | 23 (88.5) | 49 (74.2) |  | 51 (79.7) | 18 (81.8) | 24 (70.6) |  | 57 (78.1) | 17 (77.3) | 16 (72.7) |  |
| **Anti-diabetic treatment** | |  |  |  |  |  |  |  |  |  |  |  |
| *No* | 14 (45.2) | 14 (53.8) | 43 (65.2) | 0.161 | 31 (48.4) | 16 (72.7) | 21 (61.8) | 0.109 | 33 (45.2) | 17 (77.3) | 16 (72.7) | **0.007** |
| *Yes* | 17 (54.8) | 12 (46.2) | 23 (34.8) |  | 33 (51.6) | 6 (27.3) | 13 (38.2) |  | 40 (54.8) | 5 (22.7) | 6 (27.3) |  |
| **Antiplatelet agent or Anticoagulant** | | |  |  |  |  |  |  |  |  |  |  |
| *No* | 2 (6.5) | 3 (11.5) | 2 (3.0) | 0.220* | 4 (6.3) | 2 (9.1) | 1 (2.9) | 0.589* | 4 (5.5) | 2 (9.1) | 1 (4.5) | 0.853* |
| *Yes* | 29 (93.5) | 23 (88.5) | 64 (97.0) |  | 60 (93.8) | 20 (90.9) | 33 (97.1) |  | 69 (94.5) | 20 (90.9) | 21 (95.5) |  |
| **Cardiac treatment** | |  |  |  |  |  |  |  |  |  |  |  |
| *No* | 21 (67.7) | 14 (53.8) | 38 (57.6) | 0.518 | 41 (64.1) | 11 (50.0) | 20 (58.8) | 0.502 | 47 (64.4) | 10 (45.5) | 13 (59.1) | 0.283 |
| *Yes* | 10 (32.3) | 12 (46.2) | 28 (42.4) |  | 23 (35.9) | 11 (50.0) | 14 (41.2) |  | 26 (35.6) | 12 (54.5) | 9 (40.9) |  |

PSCI: Post-stroke cognitive impairment; N: Frequency; %: Percentage.
