# Supplementary figures and images for: Factors associated with cognitive impairment at 3, 6, and 12 months after the first stroke among Lebanese survivors
Source: Brain Behav. 2022 Dec 10;13(1):e2837. doi: 10.1002/brb3.2837 (PMC9847618; doi:10.1002/brb3.2837)

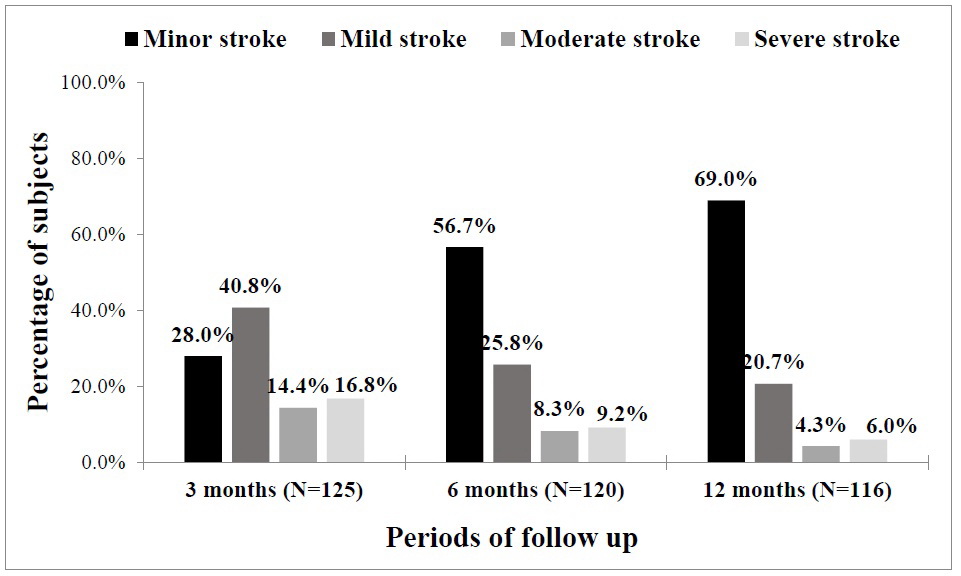

Supplement: Supplementary file 2 — Figure 1S. The severity of the stroke measured by the National Institutes of Health Stroke Scale (NIHSS). It is divided into 5 levels: 0: no stroke, 1–4: minor stroke, 5–15: moderate stroke, 15–20: moderate to severe stroke, 21–42: severe stroke. [file BRB3-13-e2837-s004.jpg]

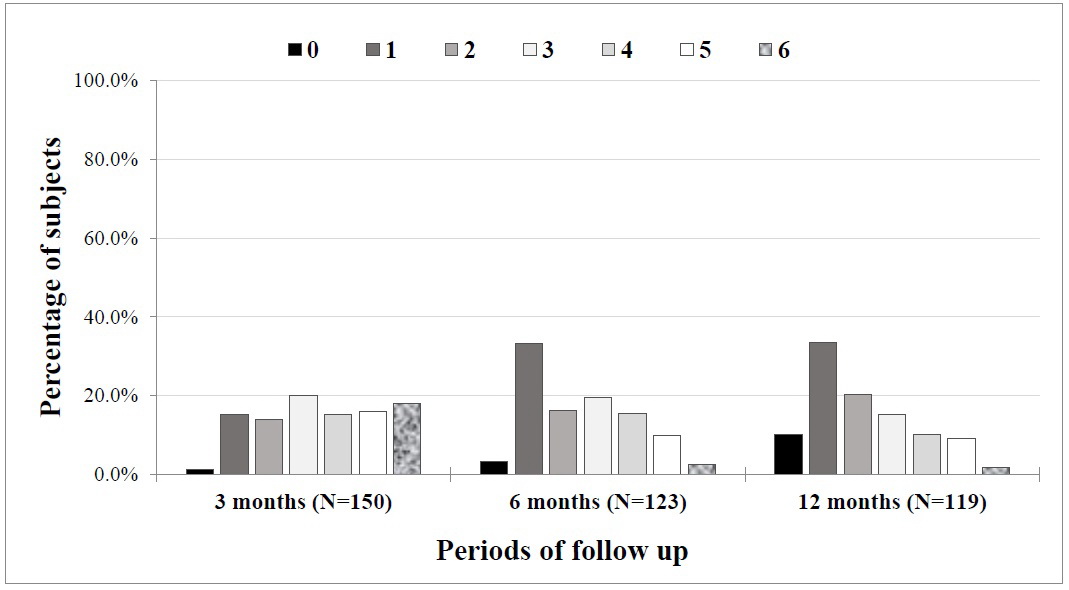

Supplement: Supplementary file 3 — Figure 2S. The degree of disability measured by the modified Rankin Scale (mRS). It is divided into 7 levels as follows: 0: no symptoms; 1: no significant disability despite symptoms; able to perform all the duties and usual activities; 2: low incapacity; unable to accomplish many previous things, but capable of taking care of its own affairs without assistance; 3: inability to moderate; needing help, but able to walk without assistance; 4: moderately serious disability; unable to walk without assistance and unable to meet physical needs without assistance; 5: serious disability; bedridden, incontinent and demanding necessary attention and constant nursing care; 6: death. [file BRB3-13-e2837-s005.jpg]

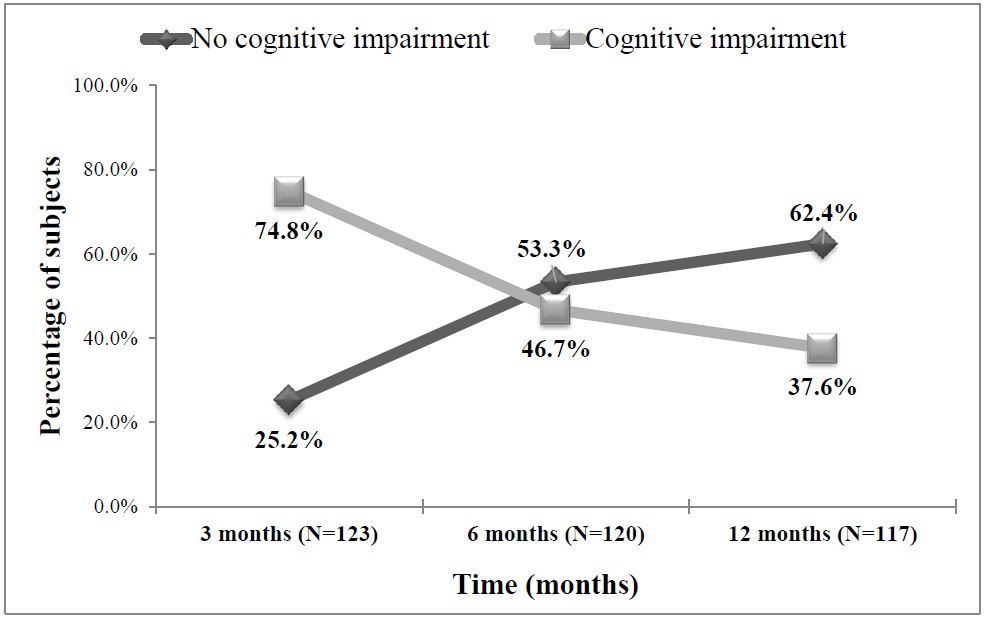

Supplement: Supplementary file 4 — Figure 3S. The rates of cognitive impairment occurring after 3, 6, and 12‐month post‐stroke. [file BRB3-13-e2837-s002.jpg]

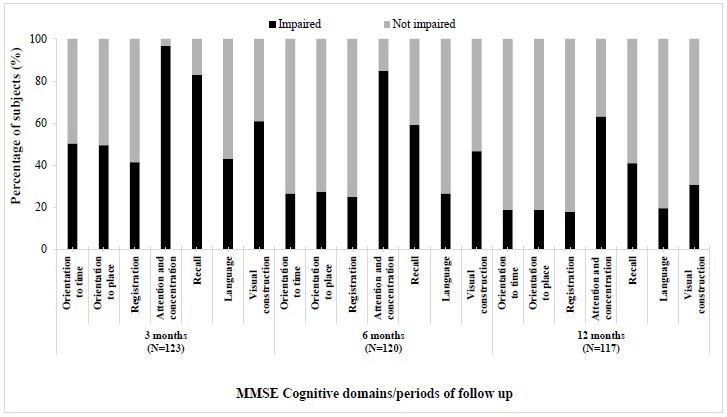

Supplement: Supplementary file 5 — Figure 4S. The percentage of subjects with/without cognitive domain impairment according to MMSE across the 3 periods of follow‐up. [file BRB3-13-e2837-s003.jpg]
